# Down-regulated miR-146a expression with increased neutrophil extracellular traps and apoptosis formation in autoimmune-mediated diffuse alveolar hemorrhage

**DOI:** 10.1186/s12929-022-00849-4

**Published:** 2022-08-26

**Authors:** Yu-Tung Hsieh, Yu-Chi Chou, Pin-Yu Kuo, Hung-Wen Tsai, Yi-Ting Yen, Ai-Li Shiau, Chrong-Reen Wang

**Affiliations:** 1 Department of Microbiology and Immunology, College of Medicine, National Cheng Kung University, Tainan, Taiwan; 2 Institute of Basic Medical Sciences, College of Medicine, National Cheng Kung University, Tainan, Taiwan; 3 Biomedical Translation Research Center, Academia Sinica, Taipei, Taiwan; 4 Departments of Pathology, National Cheng Kung University Hospital, College of Medicine, National Cheng Kung University, Tainan, Taiwan; 5 Department of Surgery, National Cheng Kung University Hospital, College of Medicine, National Cheng Kung University, Tainan, Taiwan; 6 Department of Internal Medicine, National Cheng Kung University Hospital, College of Medicine, National Cheng Kung University, Tainan, Taiwan

**Keywords:** Diffuse alveolar hemorrhage, MiR-146a, TRAF6, Neutrophil extracellular trap, NETosis, Apoptosis, IL-8, HMGB1, Systemic lupus erythematosus. intra-pulmonary delivery

## Abstract

**Background:**

Increasing evidences have suggested an important role of microRNAs (miRNAs) in regulating cell death processes including NETosis and apoptosis. Dysregulated expression of miRNAs and increased formation of neutrophil extracellular traps (NETs) and apoptosis participate in autoimmune-mediated diffuse alveolar hemorrhage (DAH), mostly associated with pulmonary capillaritis in systemic lupus erythematosus (SLE) patients. In particular, besides the inhibition of apoptosis, miR-146a can control innate and acquired immune responses, and regulate the toll-like receptor pathway through targeting TRAF6 to reduce the expression of pro-inflammatory cytokines/chemokines like IL-8, a NETosis inducer.

**Methods:**

Expression of miR-146a, TRAF6 and NETs were examined in peripheral blood neutrophils (PBNs) and lung tissues from SLE-associated DAH patients, and in neutrophils and pristane-induced DAH lung tissues from C57BL/6 mice. To assess NETs formation, we examined NETosis-related DNAs morphology and crucial mediators including protein arginine deiminase 4 and citrullinated Histone 3. Expression of miR-146a and its endogenous RNA SNHG16 were studied in HL-60 promyelocytic cells and MLE-12 alveolar cells during NETosis and apoptosis processes, respectively. MiR-146a-overexpressed and CRISPR-Cas13d-mediated SNHG16-silenced HL-60 cells were investigated for NETosis. MiR-146a-overexpressed MLE-12 cells were analyzed for apoptosis. Pristane-injected mice received intra-pulmonary miR-146a delivery to evaluate therapeutic efficacy in DAH.

**Results:**

In DAH patients, there were down-regulated miR-146a levels with increased TRAF6 expression and PMA/LPS-induced NETosis in PBNs, and down-regulated miR-146a levels with increased TRAF6, high-mobility group box 1 (HMGB1), IL-8, NETs and apoptosis expression in lung tissues. HMGB1-stimulated mouse neutrophils had down-regulated miR-146a levels with increased TRAF6, IL-8 and NETs expression. PMA-stimulated HL-60 cells had down-regulated miR-146a levels with enhanced NETosis. MiR-146a-overexpressed or SNHG16-silenced HL-60 cells showed reduced NETosis. Apoptotic MLE-12 cells had down-regulated miR-146a expression and increased HMGB1 release, while miR-146a-overexpressed MLE-12 cells showed reduced apoptosis and HMGB1 production. There were down-regulated miR-146a levels with increased TRAF6, HMGB1, IL-8, NETs and apoptosis expression in mouse DAH lung tissues. Intra-pulmonary miR-146a delivery could suppress DAH by reducing TRAF6, IL-8, NETs and apoptosis expression.

**Conclusions:**

Our results demonstrate firstly down-regulated pulmonary miR-146a levels with increased TRAF6 and IL-8 expression and NETs and apoptosis formation in autoimmune-mediated DAH, and implicate a therapeutic potential of intra-pulmonary miR-146a delivery.

**Supplementary Information:**

The online version contains supplementary material available at 10.1186/s12929-022-00849-4.

## Background

Diffuse alveolar hemorrhage (DAH) is characterized by diffuse infiltrates with bleeding in alveoli, leading to respiratory failure [1]. Most of autoimmune-mediated DAH is caused by systemic lupus erythematosus (SLE)-associated pulmonary capillaritis with neutrophilic capillary infiltration and immunoglobulin/C3 vascular deposition [1, 2]. SLE is a disease with overproduced autoantibodies due to a loss of immune tolerance [3]. Accelerated cell apoptosis and inefficient clearance results in excessive nuclear autoantigens, followed by immune complexes (ICs) formation with visceral deposition, causing DAH and glomerulonephritis (GN) [3, 4]. A dysregulated neutrophil death, described as NETosis, has been identified in SLE [4]. Overactivated neutrophils with neutrophil extracellular traps (NETs) formation and impaired removal, contributes to disease development and progression by providing autoantigens and increasing pro-inflammatory responses. Enhanced apoptosis and NETs formation have been demonstrated in the SLE-mediated DAH lungs [5, 6].

MicroRNAs (miRNAs), small noncoding RNAs binding to complementary sequences of their target mRNAs (miRNAs recognition element, MRE), modulate the expression of proteins involved in biological processes like apoptosis [7]. Particularly, miR-146a can inhibit Fas-mediated and p53-dependent apoptosis through targeting Fas-associated FADD/FAF1 and p53 co-activator/stabilizer TAF9b, respectively [8, 9]. MiRNAs have recently been shown to regulate NETs formation [10–12]. MiR-155 positively modulated the expression of protein arginine deiminase 4 (PAD4), an enzyme catalyzing histones citrullination and promoting chromatin de-condensation to form NETs [10], while interaction of neutrophils with exosomes containing miR-505 enhanced their NETosis [11]. In vitro activation of neutrophils from miR-146a deficient mouse/human had increased production of citrullinated Histone 3 (CitH3), a PAD4-catalyzed process essential for NETs formation [12]. Although miRNA-146a can regulate toll-like receptors (TLRs) and interferon (IFN) pathways by targeting TRAF6/IRAK1 and STAT1 to reduce IL-6/IL-8/IFN-α and IFN-inducible gene (ISG) expression, respectively [9], molecular mechanisms responsible for modulating TLRs-associated NETs formation remain unexplored. Furthermore, miRNAs have been identified to participate in autoimmune-mediated DAH with down-regulated miR-125a and up-regulated miR-155 pulmonary expression, while therapeutic efficacy in suppressing DAH was observed by administrating miR-125a mimics or miRNA-155 antagomirs [13, 14]. These discoveries reveal an important role of miRNAs in regulating apoptosis and NETosis and their dysregulation in autoimmune-mediated DAH.

Based on above findings, we speculated that readjustment of dysregulated pulmonary miRNAs to reduce apoptosis and NETosis has a therapeutic potential in autoimmune-mediated DAH. In this study, expression of miR-146a, TRAF6 and NETs were examined in peripheral blood neutrophils (PBNs) and lung tissues from DAH patients, and in neutrophils and pristane-induced DAH lung tissues from C57BL/6 mice. Expression of miR-146a and its endogenous RNA SNHG16 were studied in HL-60 promyelocytic and MLE-12 alveolar cells during NETosis and apoptosis, respectively. MiR-146a-overexpressed and clustered regularly interspaced short palindromic repeat (CRISPR)-Cas13d-mediated SNHG16-silenced HL-60 cells were investigated for NETosis. MiR-146a-overexpressed MLE-12 cells were analyzed for apoptosis. Pristane-injected mice received intra-pulmonary miR-146a delivery to evaluate therapeutic efficacy in DAH. Renal miR-146a expression was examined in lupus nephritis (LN) patients and a Balb/c mouse model.

## Methods

### Patients and healthy controls (HCs)

Sixty patients fulfilling the American College of Rheumatology (ACR) revised criteria for SLE [15], 54 females aged from 18 to 58 years (35.9 ± 9.6), and their age/sex-matched HCs were enrolled into this study approved by the Institutional Review Board of National Cheng Kung University Hospital (NCKUH). Clinical activities were assessed by SLEDAI-2 K with scores more than 8 as active disease [16]. DAH was diagnosed as new-onset pulmonary infiltrates, hemoglobulin drop no less than 1.5 g/dL, and histopathological or other clinical evidences [17]. LN was diagnosed by histopathological and/or laboratory findings [18]. Six patients were complicated with DAH, 5 females aged from 21 to 54 years (33.5 ± 4.8), and their age/sex-matched control groups included HCs, LN patients without DAH (LN group), and patients without DAH or LN (Nil group). DAH patients had higher SLEDAI-2 K scores than other groups, 21.0 ± 2.3 in DAH, 9.0 ± 1.0 in LN and 2.3 ± 0.6 in Nil (DAH versus LN or Nil, *p* = 0.002). Venous blood and fresh urine samples were collected from patients and HCs. Surgically biopsied lung specimens were obtained from 3 DAH patients with control pulmonary tissues from 3 non-inflammatory pneumothorax (PTX) patients.

### Purification of human or mouse cells

Human peripheral blood mononuclear cells (PBMCs) were purified from anticoagulated samples by Ficoll-Paque PLUS (GE-Healthcare, Chicago, IL, USA), and urine sediment cells (USCs) were collected from fresh specimens by centrifugation at 3000*g* for 30 min at 4 °C. PBNs were isolated by layering 5 mL anticoagulated blood over 5 mL Polymorphprep™ (AXIS-SHIELD PoC AS, Oslo, Norway) and centrifugation at 500*g* for 35 min at room temperature (RT). The granulocyte fraction was harvested, and red blood cells (RBCs) were removed through hypotonic lysis. Eight-week-old female C57BL/6 Jackson National Applied Research Laboratories (C57BL/6JNarl) mice were purchased from the National Laboratory Animal Center (Taipei, Taiwan), and housed under specific pathogen-free conditions with free access to food and water on a 12 h/12 h light–dark cycle at the NCKU Laboratory Animal Center. Animal experiments were approved by the NCKU Institutional Animal Care and Use Committee, and performed according to its guidelines. They were intraperitoneally injected with 5 mL 3% thioglycollate medium (Difco, Detroit, MI, USA), and received 5 mL phosphate-buffered saline (PBS) as lavage fluid 24 h later [19]. Peritoneal exudate cells were collected by centrifugation at 200*g* for 10 min at RT. Neutrophils were further isolated by Percoll gradient solution (Sigma-Aldrich, St. Louis, MO, USA). The purity of human or mouse neutrophils in this study was up to 95%. MiR-146a-overexpressed or SNHG16-silenecd GFP-positive cells with the expression of green fluorescence were sorted by a Moflo XDP cell sorter (Beckman Coulter, Mountain View, CA, USA).

### Generation of lentivirus (LV) vectors carrying miR-146a

The pre-microRNA expression construct containing pre-miR-146a (System Biosciences, Palo Alto, CA, USA) is a LV-based vector in which the miR-146a precursor molecule is cloned downstream of a cytomegalovirus promoter and carrying the reported gene copGFP. Recombinant LV vectors were produced by transfecting 293 T cells (American Type Culture Collection, ATCC, Manassas, VA, USA) with pre-miR-146a or pre-miRNA scramble negative control (NC) lentivector (System Biosciences), along with packaging plasmid psPAX2 and envelope plasmid pMD2.G under the calcium phosphate precipitation [20]. LV-miR-146a or LV-miR-scrambled NC (LV-miR-scr) vectors were harvested and concentrated by ultracentrifugation. Viral titers were determined in transduction unit (TU).

### Construction of pAll-EF1a-CasRx targeting SNHG16

pAll-EF1a-CasRx (16,014 bp), an all-in-one RNA-targeting CRISPR-Cas13d LV vector [21], was constructed by cloning a 6 bp human U6 terminator, a 2731 bp stuffer, a 30 bp direct repeat with an appended G (U6 promoter transcription initiation starting at the + 1 position with a G), and a 249 bp human U6 promoter into *Pac*I sites of EF1a-CasRx-2A-EFGP (Addgene, Watertown, MA, USA). Guide RNA sequences targeting human long noncoding RNA (lncRNA) SNHG16 were designed, including a NC targeting mCherry, a monomeric red fluorescent protein. A 2.7 kp stuffer was removed from pAll-EF1a-CasRx by *BsmB*I for cloning of guide sequences targeting SNHG16 after an overnight ligation reaction to create CRISPR-CasRX-SNHG16.

crRNA 01: 5ʹ-TAGAGGAACAATTAGCAGCAGAG-3ʹ.

crRNA 02: 5ʹ-TTAGAGGAACAATTAGCAGCAGA-3ʹ.

crRNA 03: 5ʹ-TTTAGAGGAACAATTAGCAGCAG-3ʹ.

crRNA 04: 5ʹ-TCTACTAAAGAAGGTACGCGCCT-3ʹ.

crRNA 05: 5ʹ-CACTTCAGAAATTCAGAGACCTC-3ʹ.

crRNA 06: 5ʹ-CCACTTCAGAAATTCAGAGACCT-3ʹ.

crRNA 07: 5ʹ-TAAAGGATTAACTCAGTCACCAG-3ʹ.

crRNA 08: 5ʹ-CCAAACAAGTTATCACACAGCAC-3ʹ.

crRNA 09: 5ʹ-ATCCAAACAAGTTATCACACAGC-3ʹ.

crRNA NC: 5ʹ-CGCCGCCGTCCTCGAAGTTCATC-3ʹ.

293 T cells (5 × 10^5^ cells/mL) in 6-well plate, were transfected with CRISPR-CasRX-SNHG16 or CRISPR-CasRX-NC for 48 h under 37 °C in the presence of polybrene (Sigma-Aldrich). Sorted GFP-positive cells were examined by quantitative real time polymerase chain reaction (qRT-PCR) analyses for SNHG16 and miR-146a expression.

### Production of sh-miR-146a-transfected HL-60 stable transfectants

Four short hairpin RNA (shRNA) sequences were designed as follows, including three targeting human miR-146a (#1, #2, #3) and one targeting luciferase, an enzyme catalyzing insect luciferin, (#4, a scramble NC).

#1, sense 5ʹ-AGTGTCAGACCTCTGAAATTA-3ʹ, antisense 5ʹ-TAATTTCAGAGGTCTGACACTTTTTT -3ʹ.

#2, sense 5ʹ-TGTCAGACCTCTGAAATTCAA-3ʹ, antisense 5ʹ-TTGAATTTCAGAGGTCTGACATTTTT-3ʹ.

#3, sense 5ʹ-CTCTGAAATTCAGTTCTTCAA-3ʹ, antisense 5ʹ-TTGAAGAACTGAATTTCAGAGTTTTT-3ʹ.

#4, sense 5ʹ- CCTAAGGTTAAGTCGCCCTCG-3ʹ, antisense 5ʹ-CGAGGGCGACTTAACCTTAGGTTTTT-3ʹ.

A 1.9 kb stuffer was removed from pLKO.1-puro (9,335 bp, National RNAi Core Facility, Academia Sinica, Taipei, Taiwan) by *Age*I and *EcoR*I for cloning shRNA sequences targeting miR-146a. To obtain recombinant LV vectors, the created pLKO.1-sh-miR-146a #1, #2, #3 and -luciferase vectors were transfected into sub-confluent 293 T cells, along with packaging psPAX2 and envelope pMD2.G plasmids by using calcium phosphate precipitation to obtain LV-sh-miR-146a #1 (Additional file 1: Fig. S1a), #2, #3 and LV-sh-luciferase, respectively [20]. The LV-sh-miR-146a #1 was chosen for further experiments based on the results of RT-PCR analyses on miR-146a levels in LV-sh-miR-146a-transfected 293 T cells (Additional file 1: Fig. S1b, 100.0 ± 2.5% for NC, 33.2 ± 1.1% for 1#, 44.6 ± 0.4% for 2#, and 57.5 ± 3.7% for 3#). We further created stable LV-sh-miR-146 #1-transfected transfectants by using puromycin selection process in sorted CRISPR-CasRX-02-transfected HL-60 cells (Additional file 1: Fig. S1c, for miR-146a, sh-luciferase versus sh-miR-146a, 100.0 ± 3.8 versus 19.2 ± 1.4%, *p* = 0.003) [5].

### qRT-PCR analyses

Total RNAs from mouse tissues and human or mouse cells were extracted by TRIzol reagent (Invitrogen, Carlsbad, CA, USA). Total RNAs from formalin-fixed, paraffin-embedded human tissues were purified by RNeasy FFPE Kit (Qiagen, Hilden, Germany). RNAs were reverse transcribed into complementary DNAs (cDNAs) by TaqMan Reverse Transcription Reagent Kit (Applied Biosystems, Foster City, CA, USA). cDNAs were used for qPCR by using the SYBR qPCR Mix Kit (TOOLS), and the amplification was performed in a RT-PCR system (Applied Biosystem). The primers sequences and melting temperature (Tm) were as follows.

Human TRAF6 (Tm 55 °C): F: 5ʹ-CCTTTGGCAAATGTCATCTGTG-3ʹ/R: 5ʹ-CTCTGCATCTTTTCATGGCAAC-3ʹ.

Mouse TRAF6 (Tm 57 °C): F: 5ʹ-GCAGTGAAAGATGACAGCGTGA-3ʹ/R: 5ʹ-TCCCGTAAAGCCATCAAGCA -3ʹ.

Human PAD4 (Tm 53 °C): F: 5ʹ-TGCTTATCCGTTAGCCGTGG-3ʹ/R: 5ʹ-GCTGTCTTGGAACACCAC-3ʹ.

Human HMGB1 (Tm 58 °C): F: 5ʹ-GCGAAGAAACTGGGAGAGATGTG-3ʹ/R: 5ʹ-GCATCAGGCTTTCCTTTAGCTCG-3ʹ.

Human IL-6 (Tm 53 °C): F: 5ʹ-ACTCACCTCTTCAGAACGAATTG-3ʹ/R: 5ʹ-CATCTTTGGAAGGTTCAGGTTG-3.

Mouse IL-6 (Tm 55 °C): F: 5ʹ-CTGCAAGAGACTTCCATCCAG-3ʹ/R: 5ʹ-AAGTGGTATAGACAGGTCTGTTGG-3ʹ.

Human IL-8 (Tm 58 °C): F: 5ʹ-GAGAGTGATTGAGAGTGGACCAC-3ʹ/R: 5ʹ-CACAACCCTCTGCACCCAGTTT-3ʹ.

Mouse IL-8 (Tm 57 °C): F: 5ʹ-TGCATGGACAGTCATCCACC-3ʹ/R: 5ʹ-ATGACAGACCACAGAACGGC-3ʹ.

Human IFN-α (Tm 62 °C): F: 5ʹ-AATCTCTCCTTCCTCCTGTCTGATG-3ʹ/R: 5ʹ-TCTGACAACCTCCCAGGCACA-3ʹ.

Mouse IFN-α (Tm 57 °C): F: 5ʹ-TGTCTGATGCAGCAGGTGG-3ʹ/R: 5ʹ-AAGACAGGGCTCTCCAGAC-3ʹ.

Human MX-1 (Tm 58 °C): F: 5ʹ-GGACTGCGAGGATGATG-3ʹ/R: 5ʹ-CGCCAGCTCATGTGCATCT-3ʹ.

Mouse MX-1 (Tm 60 °C): F: 5ʹ-TGGACATTGCTACCACAGAGGC-3ʹ/R: 5ʹ-TTGCCTTCAGCACCTCTGTCCA-3ʹ.

Human SNHG16 (Tm 57 °C): F: 5ʹ-CAGAATGCCATGGTTTCCCC-3ʹ/R: 5ʹ-TGGCAAGAGACTTCCTGAGG -3ʹ.

Mouse SNHG16 (Tm 61 °C): F: 5ʹ-TGACTCGGAAGGGTGCCTGTG-3ʹ/R: 5ʹ-AATCTGCCACTTAGCACACCCCTC-3ʹ.

Human GAPDH (Tm 54 °C): F: 5ʹ-ACTTCAACAGCGACACCCACT-3ʹ/R: 5ʹ-GCCAAATTCGTTGTCATACCAG-3ʹ.

Mouse GAPDH (Tm 56 °C): F: 5ʹ-GTTGTCTCCTGCGACTTCAACA-3ʹ/R: 5ʹ-TTGCTGTAGCCGTATTCATTGTC-3ʹ.

All mRNA levels were normalized to GAPDH by ΔCt method [20]. For analyzing human or mouse miR-146a levels, total RNAs were reverse transcribed by a TaqMan MicroRNA reverse transcriptase kit (Applied Biosystems) in Smart Cycler (Cepheid, Sunnyvale, CA, USA). Quantitative levels of miR-146a were analyzed with RNU6B small RNA (Applied Biosystems) as an endogenous control. The average levels of HCs, control human tissues, control mouse tissues or cells on day 0, cell lines without stimulation, miR-146a-scr-, CRISPR-CasRX-NC-, or sh-luciferase-transfected cells were determined as 100%.

### Preparation of hydrophilic pristane

Although to study in vitro biological responses of pristane is prohibited by its hydrophobicity, this barrier can be removed by forming a hydrophilic inclusion complex with β-cyclodextrin (βCD), a D-glucose oligomer with hydrophilic surface, to effectively deliver pristane in vitro [22]. A bolus of 2 mM pristane (Sigma-Aldrich) was pipetted into 4 mM solution of βCD (Sigma-Aldrich) and stirred at RT for 4 d [5]. The crystalline complexes which precipitated out of solution were washed twice in PBS and stored at 4 °C [22]. The concentrations were measured by UV spectrometry with optical densities at 254 nm.

### NETs formation in human or mouse cells

PBNs (5 × 10^5^ cells/mL) were allowed to adhere to poly-L-lysine (Sigma-Aldrich) coated 24-well plate in the presence of 250 ng/mL phorbol 12-myristate 13-acetate (PMA, Sigma-Aldrich) or 3 μg/mL lipopolysaccharide (LPS) from *Pseudomonas aeruginosa* 10 (Sigma-Aldrich) [23] for 4 h under 37 °C. Mouse neutrophils (10^6^ cells/mL) were cultured in 3.5 cm dish for 30 min under 37 °C. The attached neutrophils were incubated under the same condition with different concentrations of βCD-pristane or mouse high-mobility group box 1 (HMGB1, Atlantis Bioscience, Singapore, Republic of Singapore) for 4 h under 37 °C, and with the LPS stimulation as a positive control (PC). Human promyelocytic cells (HL-60, ATCC) were cultured with 10^6^ cells/mL in 3.5 cm dish in the presence of 1.25% dimethyl sulfoxide (DMSO, Sigma-Aldrich) for 5 d under 37 °C to induce differentiated HL-60 (dHL-60) cells. These cells were further cultured in the presence of 50 ng/mL PMA with serum-free X-VIVO 15 medium (Lonza, Basel, Switzerland) for 4 h under 37 °C. After culture, human or mouse cells were stained with Sytox Green (Thermo Fisher Scientific, Waltham, MA, USA) to detect DNAs under fluorescence microscopy. Their morphology was categorized into lobulated neutrophils, de-lobulated neutrophils, diffused NETs or spread NETs category [24]. These cells were further subjected to qRT-PCR analyses. Their culture supernatants and cells lysates were quantified by enzyme-linked immunosorbent assay (ELISA) for the levels of CitH3 and PAD4 (Cayman, Ann Arbor, Michigan, USA), respectively.

### NETs formation in miR-146a-overexpressed, SNHG16-silenced or miR-146a- silenced HL-60 cells

HL-60 cells (10^6^ cells/mL) in 3.5 cm dish were transfected with LV-miR-146a, LV-miR-scr, CRISPR-CasRX-SNHG16 or CRISPR-CasRX-NC for 48 h under 37 °C in the presence of polybrene. GFP-positive cells were sorted and examined by qRT-PCR analyses. Sorted or mock cells were cultured in the presence of 1.25% DMSO for 5 d to induce dHL-60 cells. In addition, miR-146a #1- or luciferase-silenced CRISPR-CasRX-02-transfected HL-60 stable transfectants were cultured in the same condition for 5 d. These cells were then stimulated with 50 ng/mL PMA under serum-free X-VIVO 15 medium for 4 h, stained with Sytox Green, and observed under fluorescence microscopy. Their culture supernatants and cells lysates were examined for CitH3 and PAD4 levels, respectively. Un-transfected dHL-60 cells were stimulated in the presence of 100 μM chloramidine (Cl-amidine, Cayman), a PADs inhibitor to inhibit NETosis as a PC.

### Apoptosis induction in MLE-12 cells

Mouse alveolar cells (MLE-12, ATCC) were seeded with 1 × 10^6^ cells/mL in 3.5 cm dish in the presence of different concentrations of doxorubicin (Dox, TTY Biopharm, Taipei, Taiwan), a DNA damage inducer to trigger p53-dependent cell apoptosis [25], or different concentrations of βCD-pristane for 24 h under 37 °C. After stimulation, these cells were subjected to qRT-PCR analyses. Their culture supernatants were assessed for HMGB1 concentrations by ELISA (LSBio, Seattle, WA, USA). After stimulation, cells were stained with PE-Annexin V (BD Pharmingen, San Diego, CA, USA) and 7-amino-actinomycin D (7-AAD, BD Pharmingen). Annexin V-positive and 7-AAD-negative cells were defined as apoptosis, and average apoptotic percentages without stimulation was defined as apoptotic cell ratios 1.0 [5, 26]. Alternatively, these cells were stained by terminal deoxynucleotidyl transferase dUTP nick end labeling (TUNEL) detection cocktail (Promega, Madison, WI, USA) with cell nuclei counterstained by DAPI (Sigma-Aldrich), and observed under confocal microscopy with a FluoView FV3000 (Olympus, Tokyo, Japan).

### Apoptosis induction in miR-146a-overexpressed MLE-12 cells

MLE-12 cells (1 × 10^6^ cells/mL) in 3.5 cm dish were transfected with LV-miR-scr or LV-miR-146a for 48 h under 37 °C in the presence of polybrene. GFP-positive cells were sorted and examined by qRT-PCR analyses. Sorted cells were stimulated with 1 µM Dox for 24 h under 37 °C. After stimulation, these cells were incubated with Hoechst 33,258 (Thermo Fisher Scientific) and Annexin V Alexa Fluor 647 conjugate (BioLegend, San Diego, CA, USA), and observed under confocal microscopy. Culture supernatants were examined for HMGB1 levels. In addition, un-transfected MLE-12 cells were stimulated in the presence of 10 µM Z-VAD-FMK (Selleckchem, Houston, TX, USA), a pan-caspase inhibitor to inhibit apoptosis as a PC.

### Pristane-induced mouse DAH or LN model

Eight-week-old female C57BL/6JNarl mice received intraperitoneal injection of 0.5 mL pristane to induce DAH, and their controls were injected with 0.5 mL PBS [5]. They were sacrificed on day 0, 4, 9 and 14 to obtain their lungs and spleen. Anticoagulated blood samples were measured for neutrophil, RBC numbers, hematocrit (Hct) and hemoglobulin (Hb) levels by a blood cell analyzer (Scil Vet Focus 5, Scil Animal Care, Viernheim, Germany).

Eight-week-old female BALB/cJNarl mice were purchased from the National Laboratory Animal Center, and received intraperitoneal injection of 0.5 mL pristane to induce LN, while their controls were injected with 0.5 mL PBS [26]. Urine specimens were collected for measuring proteinuria (protein/creatinine) by test strips (Arkray, Edina, MN, USA). The results were determined by semi-automated urine chemistry analyzer (Arkray RT-4010) at month 0, 1, 3, 5 and 6. Blood samples from mice were examined for the presence of anti-dsDNA levels with an ELISA kit (Alpha Diagnosis, San Antonio, TX, USA) at month 0, 1, 3, 5 and 6. Their kidneys were removed for measuring miR-146a levels at month 0, 1, 3, 5, and 6, and for analyzing renal histopathology at month 6.

### Immunoblotting assay

Mouse lung tissue homogenates were separated by electrophoresis on 10 to 15% SDS-PAGE, transferred on PVDF membranes (Merck Millipore, Burlington, MA, USA), blocked in 5% of non-fat dry milk, and incubated with primary antibodies including anti-CitH3 (1:1000, citrulline R2 + R8 + R17, Abcam, Cambridge, UK), anti-TRAF6 (1:1000, Santa Cruz), anti-IRAK-1 (1:1000, Santa Cruz), anti-PAD4 (1:1000, Abcam), anti-HMGB1 (1:1000, Abcam) or anti-β-actin antibodies (1:5000, Sigma-Aldrich) at 4 °C for 18 h. After washing, the membranes were incubated with HRP-conjugated secondary antibodies (1:10,000, Jackson Immunoresearch, West Grove, PA, USA) at RT for 2 h. Signal expression of protein-antibody complexes was detected by ECL system (Amersham Pharmacia Biotech, Buckinghamshire, UK) and visualized with Biospectrum imaging system (UVP, Upland, CA, USA). Relative protein expression levels were measured by Image J (National Institute of Health, Bethesda, MD, USA).

### Intra-tracheal LV-miR-146a delivery and therapeutic evaluation

Mice received 2 × 10^9^ TU/mL of LV-miR-146a or LV-miR-scr by intra-tracheal delivery of fluid bolus into the posterior oropharynx above the tracheal entrance [5], and intraperitoneal injection of 0.5 mL pristane. DAH was evaluated according to gross and histopathological findings with three categories including no, partial and complete hemorrhage on day 14 [5].

### Histopathological, TUNEL and immunofluorescence staining

Removed lung and kidney tissues were fixed in 10% buffered formalin overnight, and embedded in paraffin. Lung tissues were cut into 5 µm sections, and stained with hematoxylin and eosin (H&E). Paraffin-embedded sections were de-paraffinized in xylene, dehydrated in ethanol and rehydrated in distilled water. To determine GN, mouse renal tissues were analyzed by Periodic acid-Schiff (PAS) staining [26]. For TUNEL staining, de-paraffinized lung sections were treated by proteinase K to reactivate antigens, re-fixed by 4% formaldehyde, incubated with equilibrate buffer, and finally labelled by TUNEL detection cocktail [5, 26]. TUNEL-positive cells were determined by averaging the number from 3 fields (× 400) of positively stained cells with the highest density in each section. Cell nuclei were counterstained with DAPI. Fluorescence was detected by confocal microscopy. For detecting the expression of CitH3, de-paraffinized human or mouse lung sections were stained with anti-CitH3 antibodies, followed by Alexa Fluor 488-conjugated antibodies (Thermo Fisher Scientific). Cell nuclei were counterstained with Hoechst 33258. Fluorescence was detected by confocal microscopy.

### Statistical analyses

Data are expressed as the mean ± standard error of mean (SEM). MiR-146a levels, TRAF6 levels, morphology percentages or CitH3 concentrations between patients and HCs or different patient groups were analyzed by Mann–Whitney U test. Correlation analysis was performed by Spearman correlation coefficient test. Complete hemorrhage frequencies between LV-miR-scr- and LV-miR-146a-treated mice were compared by Fisher’s exact test. Differences in other analyses were determined by Student’s t test. *p* values less than 0.05 were considered significant in this study with symbols as * for *p* < 0.05, ** *p* < 0.01 and *** for *p* < 0.001.

## Results

### Down-regulated miR-146a expression in PBNs from DAH patients

**Fig. 1:**
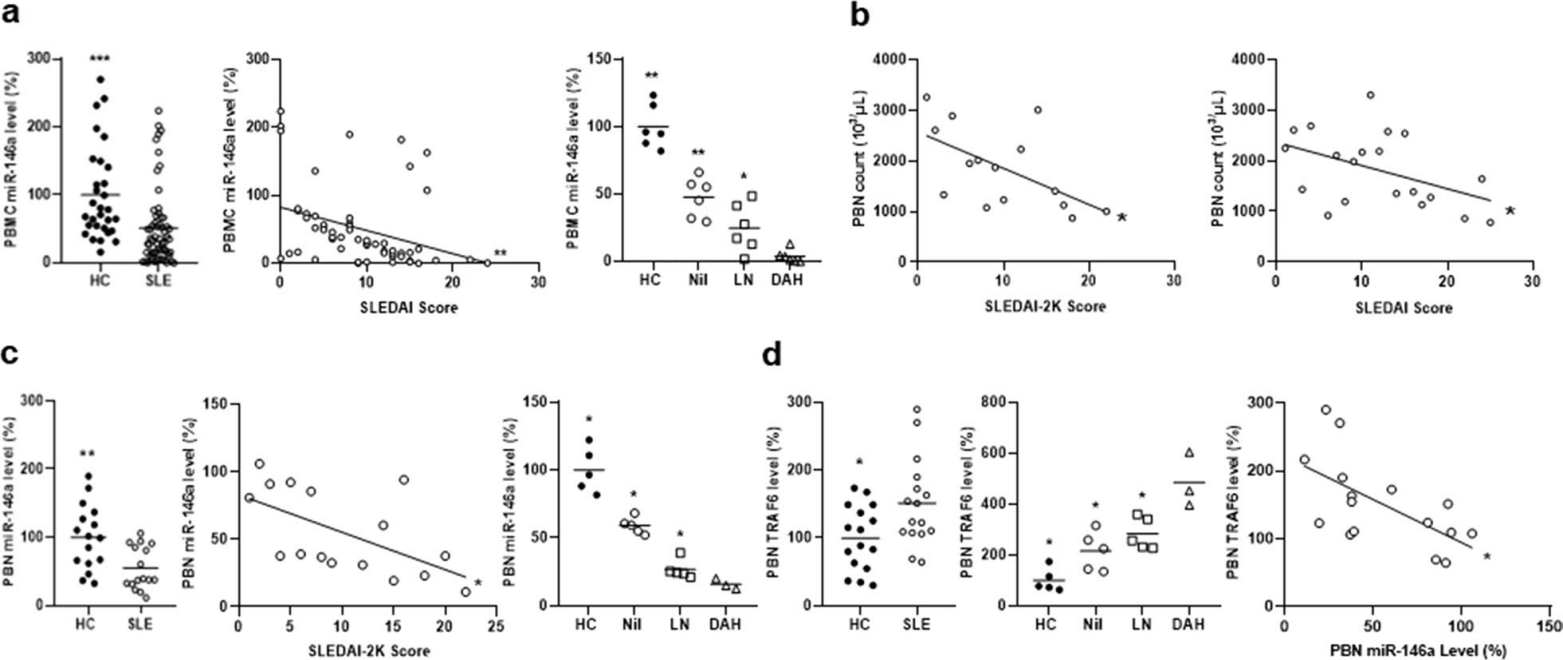
Down-regulated miR-146a expression in PBNs from DAH patients. **a** Left, miR-146a levels in PBMCs from HCs and SLE patients. Middle, a negative correlation between miR-146a levels in PBMCs and activity scores. Right, miR-146a levels in PBMCs from HCs, Nil, LN and DAH patients. **b** A negative correlation between PBN counts and activity scores. Left, newly diagnosed and treatment-naïve SLE patients. Right, anti-neutrophil cytoplasmic antibody-positive SLE patients. **c** Left, miR-146a levels in PBNs from HCs and SLE patients. Middle, a negative correlation between miR-146a levels in PBNs and activity scores. Right, miR-146a levels in PBNs from HCs, Nil, LN and DAH patients. **d** Left, TRAF6 levels in PBNs from HCs and SLE patients. Middle, TRAF6 levels in PBNs from HCs, Nil, LN and DAH patients. Right, A negative correlation between TRAF6 and miR-146a levels in PBNs from SLE patients. Values are mean ± SEM. Horizontal lines are mean values. Patient numbers, n = 60 for PBMCs, n = 15 (left) and n = 20 (right) for PBN counts, n = 16 for PBNs. * *p* < 0.05, ** *p* < 0.01, *** *p* < 0.001

At first, PBMCs from SLE patients and HCs were examined for miR-146a expression. Lower levels were found in SLE patients than HCs (Fig. 1a, left, 51.4 ± 7.3% versus 100.0 ± 12.4%, p < 0.001). DAH patients had lower miR-146a levels than LN patients, Nil patients or HCs (Fig. 1a, middle, *p* = 0.026 for LN, *p* = 0.002 for Nil or HCs). A negative correlation was found between miR-146a levels and activity scores (Fig. 1a, right, r = − 0.334, *p* = 0.009).

In the NCKUH hospitalized SLE cohort [2, 17], PBN counts in 15 newly diagnosed, treatment-naïve patients including 5 with DAH, were negatively correlated with activity scores (Fig. 1b, left, r = − 0.578, *p* = 0.029). Furthermore, in 20 anti-neutrophil cytoplasmic antibody-positive patients including 3 with DAH, their PBN counts were correlated inversely with SLEDAI-2 K scores (Fig. 1b, right, r = − 0.454, *p* = 0.044). These finding implied a pathogenic role of neutrophil death in the development and progression of SLE and its DAH manifestation by providing a source of autoantigens [4]. Lower miR-146a levels were found in PBNs from SLE patients than HCs (Fig. 1c, left, 54.9 ± 7.9% versus 100.0 ± 11.9%, *p* = 0.005). A negative correlation was found between miR-146a levels and activity scores (Fig. 1c, middle, r = − 0.589, *p* = 0.016). DAH patients had lower miR-146a levels than LN patients, Nil patients or HCs (Fig. 1c, right, *p* = 0.036 in all).

The levels of TRAF6, a miR-146a target molecule, in PBNs were higher in SLE patients than HCs (Fig. 1d, left, 151.3 ± 16.2% versus 100.0 ± 12.0%, *p* = 0.038). DAH patients had higher TRAF6 levels than LN patients, Nil patients or HCs (Fig. 1d, middle, *p* = 0.036 in all). A negative correlation was found between TRAF6 and miR-146a levels in SLE patients (Fig. 1d, right, r = − 0.616, *p* = 0.011).

### Increased NETs formation in PBNs from DAH patients

**Fig. 2:**
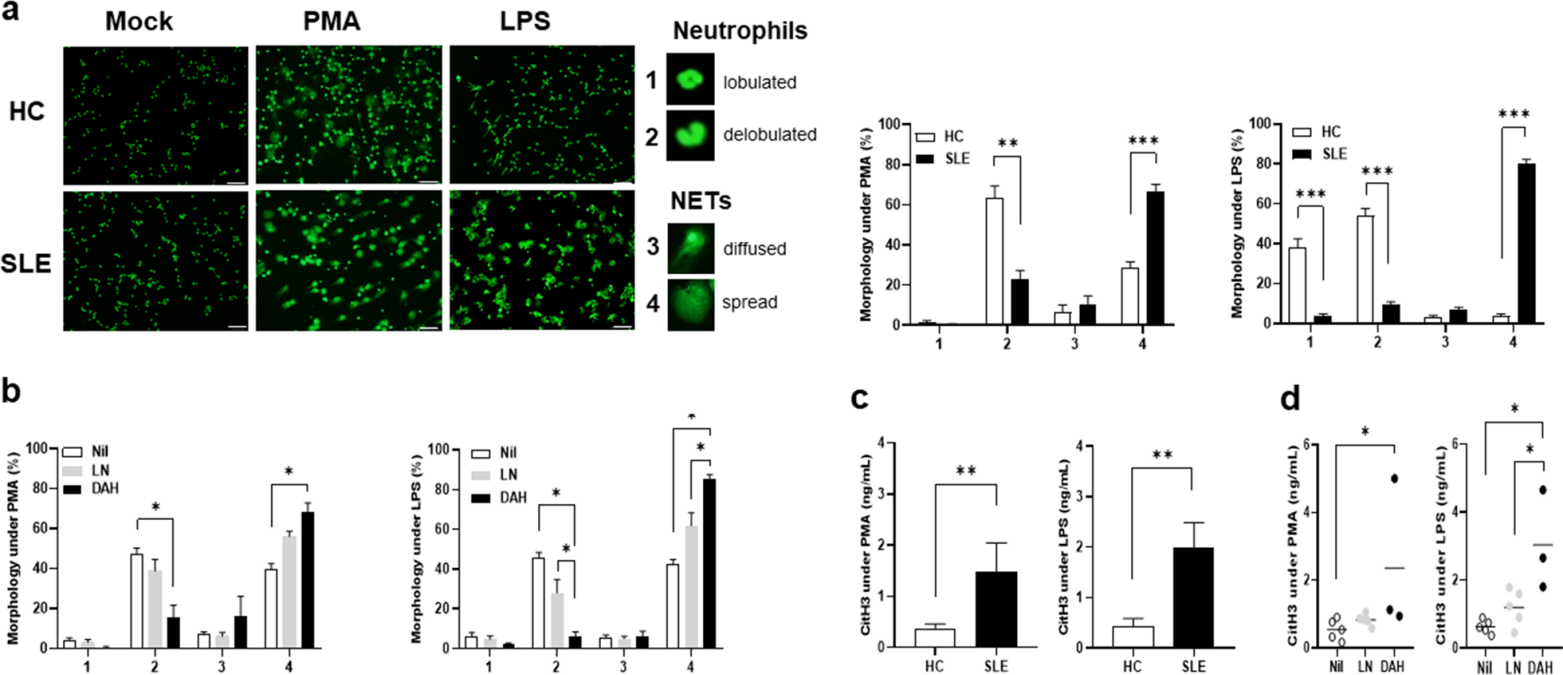
Increased NETs formation in PBNs from DAH patients. **a** PBNs from SLE patients or HCs stimulated with PMA or LPS and stained with Sytox Green to detect DNAs morphology. Left, representative photographs from a HC and a SLE patient. Scale bar = 50 µm, magnification × 200. Right, quantification of NETs formation in HCs and SLE patients. **b** Quantification of NETs formation by PMA or LPS stimulation in DAH, LN and Nil patients. **c** CitH3 levels in PMA or LPS-stimulated supernatants from HCs and SLE patients. **d** CitH3 levels in PMA or LPS-stimulated supernatants from DAH, LN and Nil patients. Values are mean ± SEM. Horizontal lines are mean values. Patient numbers, n = 7 for SLE, n = 3 for DAH, n = 5 for LN, n = 5 for Nil. * *p* < 0.05, ** *p* < 0.01, *** *p* < 0.001

PBNs from different patient groups and HCs were stimulated with PMA or LPS to induce NETs formation. SLE patients had higher percentages of spread NETs than age/sex-matched HCs (Fig. 2a, for PMA, 66.8 ± 3.4 versus 28.9 ± 2.8%, *p* < 0.001, for LPS, 79.9 ± 2.1 versus 3.8 ± 1.1%, *p* < 0.001). DAH patients had higher percentages of spread NETs than LN or Nil patients (Fig. 2b, DAH versus Nil, for PMA, 68.1 ± 4.6 versus 39.3 ± 3.2%, *p* = 0.036, for LPS, 85.4 ± 2.0 versus 42.6 ± 2.3%, *p* = 0.036, DAH versus LN, for PMA, 68.1 ± 4.6 versus 58.1 ± 3.8%, *p* = 0.143, for LPS, 85.4 ± 2.0 versus 61.9 ± 6.5%, *p* = 0.036). Furthermore, culture supernatants under PMA or LPS stimulation were examined for CitH3 levels. SLE patients had higher levels than HCs (Fig. 2c, for PMA 1.48 ± 0.56 versus 0.35 ± 0.11 ng/mL, *p* = 0.001, for LPS, 1.99 ± 0.49 versus 0.43 ± 0.16 ng/mL, *p* = 0.007). DAH patients had higher levels than LN or Nil patients (Fig. 2d, DAH versus Nil, for PMA, 2.36 ± 1.32 versus 0.54 ± 0.14 ng/mL, *p* = 0.036, for LPS, 3.03 ± 0.85 versus 0.63 ± 0.09 ng/mL, *p* = 0.036, DAH versus LN, for PMA, 2.36 ± 1.32 versus 0.83 ± 0.08 ng/mL, *p* = 0.071, for LPS, 3.03 ± 0.85 versus 1.19 ± 0.24 ng/mL, *p* = 0.036). These data suggested greater NETs formation in PBNs from DAH patients with lower miR-146a expression.

### Down-regulated pulmonary miR-146a expression with increased NETs and apoptosis formation in DAH patients

**Fig. 3:**
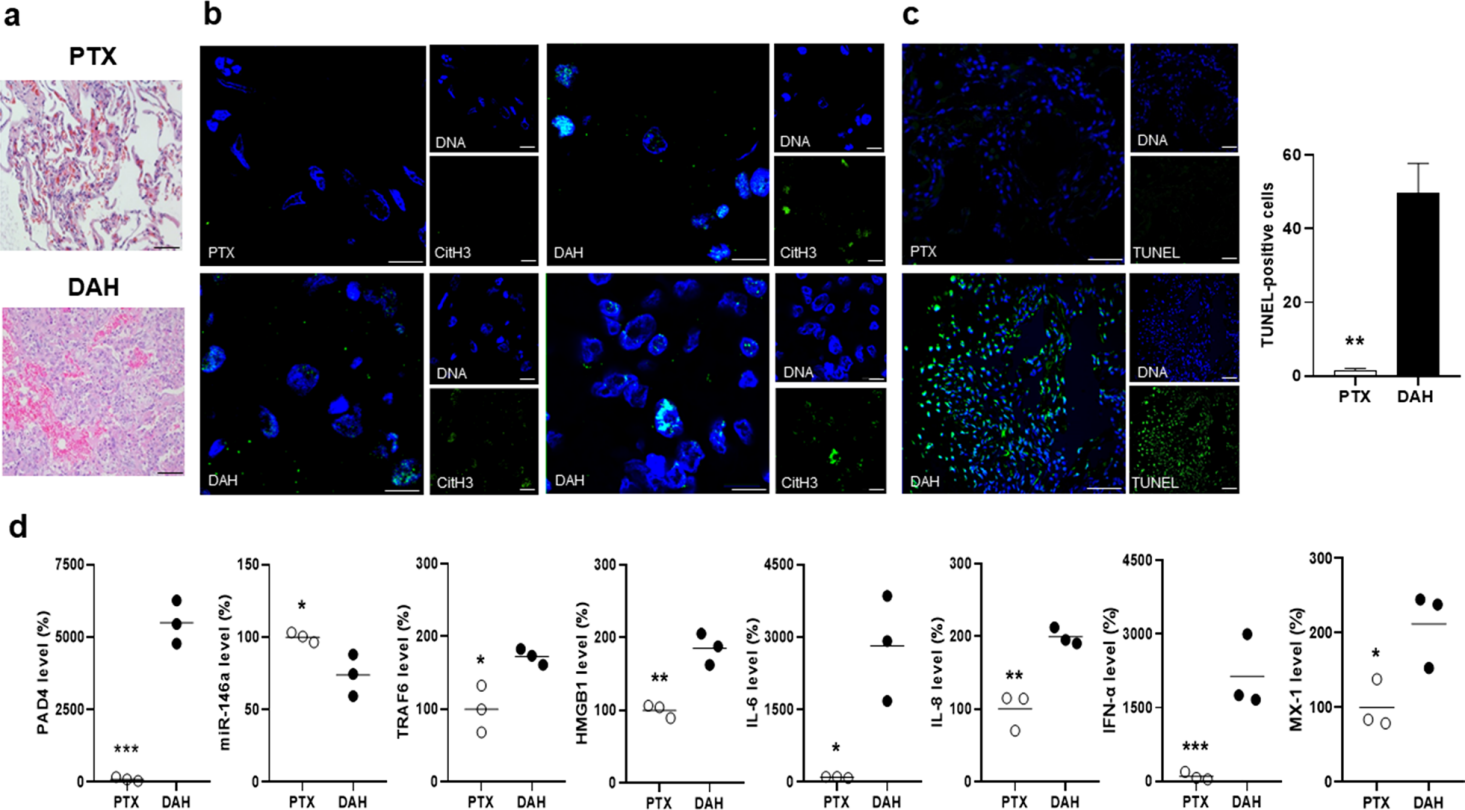
Down-regulated pulmonary miR-146a expression with increased NETs and apoptosis formation in DAH patients. **a** Representative H&E staining of lung tissues from a PTX and a DAH patient. Scale bar = 40 µm, magnification × 200. **b** Representative CitH3 IF staining (green) from a PTX and 3 DAH patients. Cell nuclei counterstained with Hoechst 33,258 (blue). Scale bar = 10 µm, magnification × 1000. **c** Left, representative TUNEL IF staining (green) from a PTX and a DAH patient. Cell nuclei counterstained with DAPI (blue). Scale bar = 25 µm, magnification × 400. Right, quantification of TUNEL-positive cell numbers in lung tissues. **d** Expression levels of PAD4, miR-146a, TRAF-6, HMGB1, IL-6, IL-8, IFN-α and MX-1 in lung tissues from 3 PTX and 3 DAH patients. Values are mean ± SEM. Horizontal lines are mean values. * *p* < 0.05, ** *p* < 0.01, *** *p* < 0.001

Figure 3a shows H&E-stained lung tissues from DAH and PTX patients. Since there was down-regulated miR-146a expression with increased NETs formation in PBNs from DAH patients, we further examined their lung tissues for the expression of miR-146a, TRAF6, CitH3, PAD4, HMGB1, IL-6, IL-8, IFN-α and MX-1 (an ISG). Distinct expression of CitH3 colocalized with DNAs, in favor of NETs, was identified in DAH but not PTX lung tissues (Fig. 3b). Higher numbers of TUNEL-positive cells were found in lung tissues from DAH than PTX patients (Fig. 3c, 49.6 ± 8.1 versus 1.5 ± 0.6, *p* = 0.004). Pulmonary PAD4 levels were higher in lung tissues from DAH than PTX patients (Fig. 3d, 5,499.0 ± 428.9 versus 100.0 ± 39.2%, *p* < 0.001), suggestive of increased NETs formation. Down-regulated miR-146a and up-regulated TRAF-6 expression were found in lung tissues from DAH patients (*p* = 0.018 for miR-146a, *p* = 0.021 for TRAF-6). There were increased levels of HMGB1, IL-6, IL-8, IFN-α and MX-1 in DAH lung tissues (*p* = 0.003 for HMGB1, *p* = 0.013 for IL-6, *p* = 0.003 for IL-8, *p* = 0.004 for IFN-α, *p* = 0.033 for MX-1). These results implicated down-regulated miR-146a levels with increased expression of TRAF6, HMGB1, IL-6 and IL-8, and formation of NETs and apoptosis in the human DAH lungs.

### Reduced NETosis in miR-146a-overexpressed, SNHG16-silenced or miR-146a- silenced HL-60 cells

**Fig. 4:**
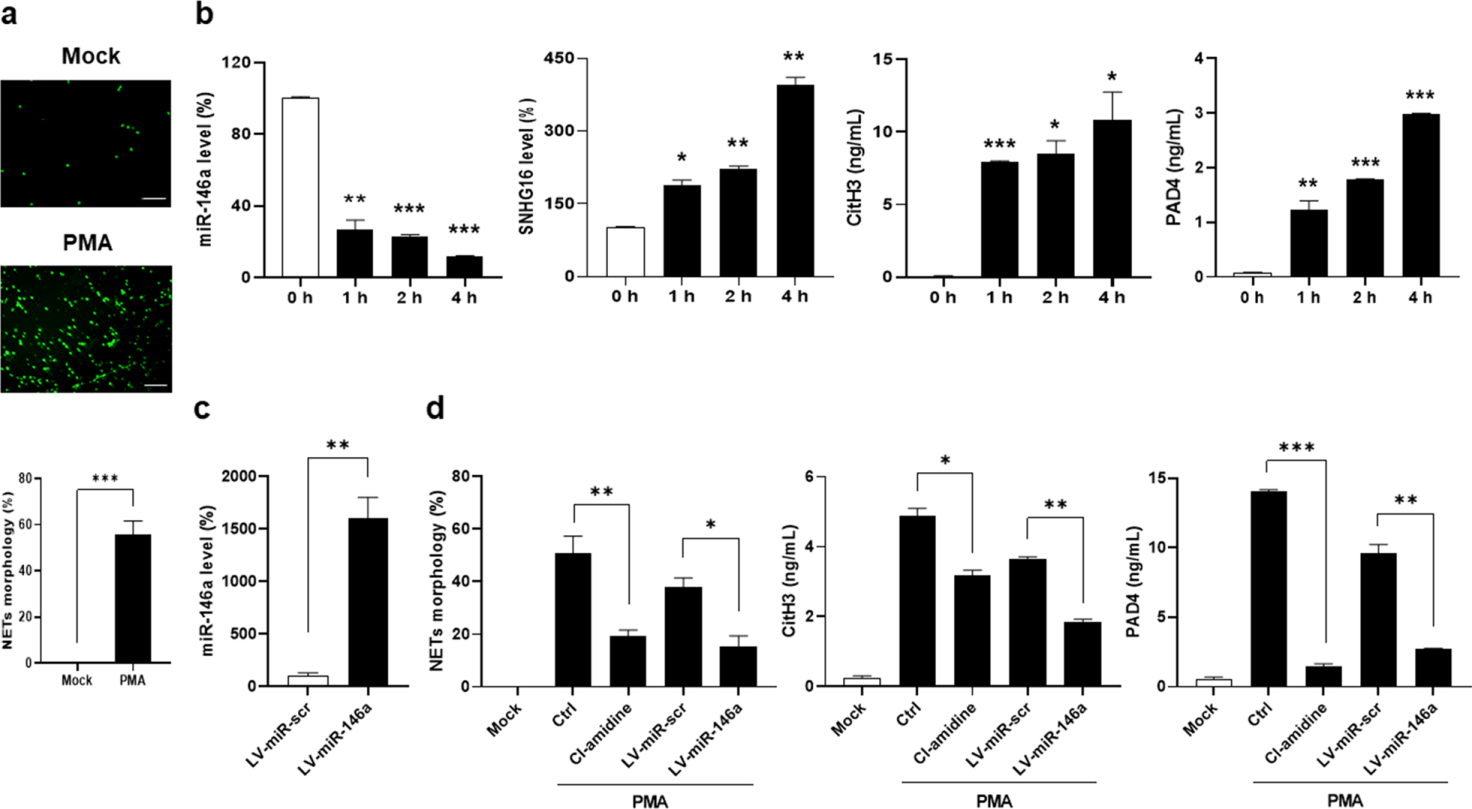
Reduced NETosis in miR-146a-overexpressed dHL-60 cells. **a** dHL-60 cells stimulated with PMA and stained with Sytox Green to detect DNAs morphology. Upper, Representative photographs from mock and PMA stimulation. Scale bar = 60 µm, magnification × 200. Lower, quantification of NETosis percentages with the diffused/spread NETs morphology. **b** MiR-146a (left), SNHG16 (middle left), CitH3 (middle right) and PAD4 (right) levels in dHL-60 cells stimulated with PMA for different times. **c** MiR-146a levels in LV-miR-146a-transfected HL-60 cells. **d** Quantification of NETosis percentages (left), CitH3 (middle) and PAD4 (right) levels in miR-146a-overexpressed dHL-60 cells under PMA stimulation. The presence of Cl-amidine as a PC. Values are mean ± SEM. All results in this figure were representative of 3 independent experiments with similar findings. * *p* < 0.05, ** *p* < 0.01, *** *p* < 0.001

There were increased percentages of diffused/spread NETs morphology (Fig. 4a), and time-dependent down-regulated miR-146a and up-regulated CitH3/PAD4 expression in PMA-stimulated dHL-60 cells (Fig. 4b). In particular, miR-146a expression has been reported to be regulated by SNHG16, a pro-apoptotic lncRNA [27], and time-dependent up-regulated SNHG16 expression was found in PMA-stimulated dHL-60 cells (Fig. 4b). MiR-146a-overexpressed HL-60 cells had increased miR-146a levels (Fig. 4c, miR-146a versus miR-scr, 1605.7 ± 199.3 versus 100.0 ± 29.6%, *p* = 0.009). After PMA stimulation, there were lower NETs percentages, CitH3 and PAD4 levels in miR-146a-overexpressed dHL-60 cells (Fig. 4d, miR-146a versus miR-scr, for NETs, 17.0 ± 4.4 versus 42.3 ± 3.8%, *p* = 0.012, for CitH3, 1.85 ± 0.08 versus 3.65 ± 0.06 ng/mL, *p* = 0.003, for PAD4, 2.71 ± 0.06 versus 9.64 ± 0.60 ng/mL, *p* = 0.008).

**Fig. 5:**
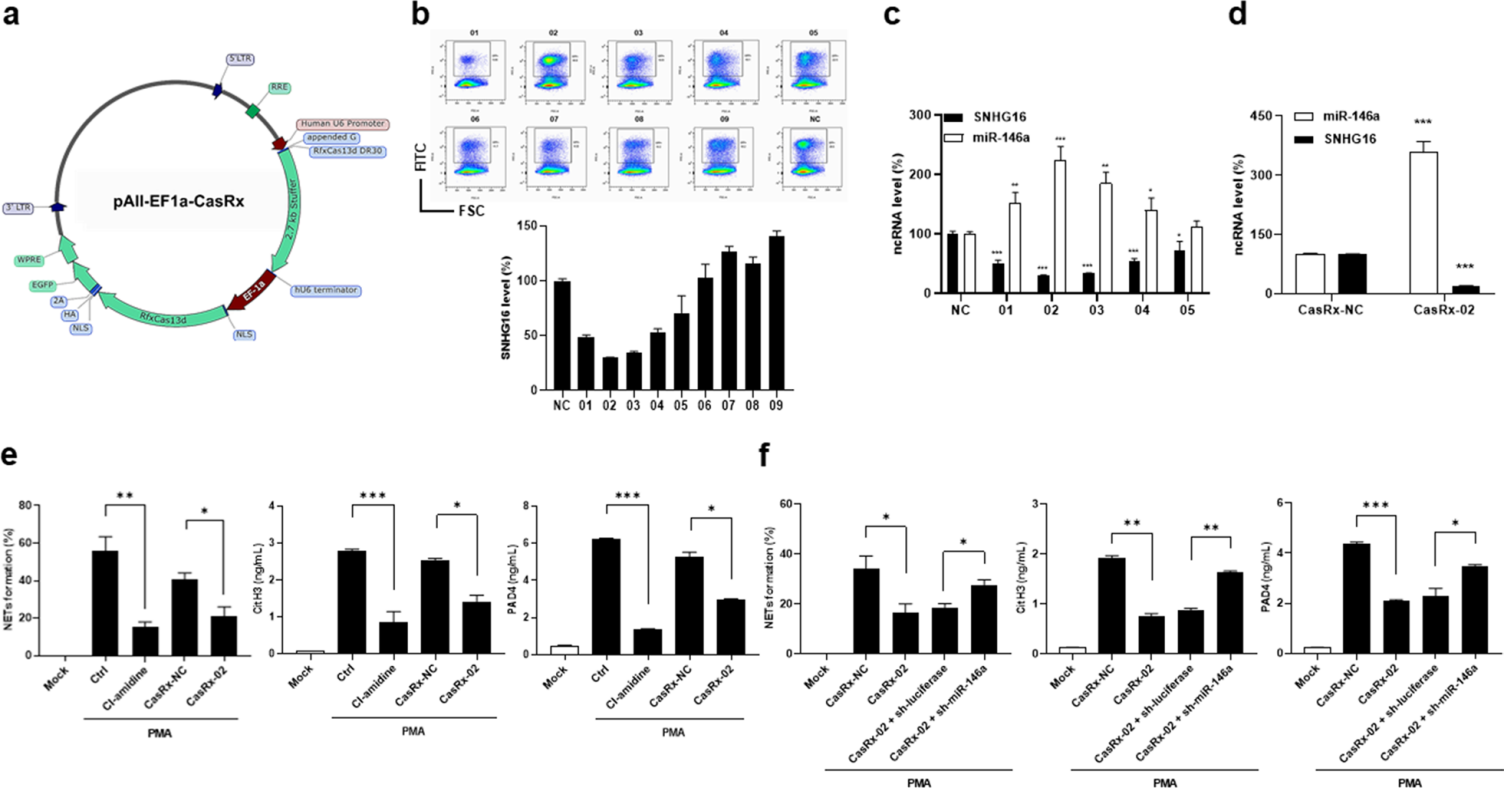
Reduced NETosis in SNHG16- or miR-146a-silenced HL-60 cells. **a** Map of pAll-EF1a-CasRx (16,014 bp in length) with an EGFP insertion, a Cas13d domain, a 2.7 k bp stuffer and a 30 bp DR with an appended G. **b** Upper, flow cytometric graphs of sorted GFP-positive CasRx-transduced cell portion for qRT-PCR analyses including CasRx-01 to 09- and CasRx-NC-transfected 293 T cells. Lower, SNHG16 levels for silencing efficacy in CasRx-01 to 09- and CasRx-NC-transfected 293 T cells. **c** SNHG16 and miR-146a levels in CasRx-01 to 05- and CasRx-NC-transfected 293 T cells. **d** SNHG16 and miR-146a levels in CasRx-02-transfected HL-60 cells. **e** Quantification of NETs formation percentages (left), CitH3 levels (middle), and PAD4 levels (right) in SNHG16-silenced dHL-60 cells under PMA stimulation. **f** Quantification of NETs formation percentages (left), CitH3 levels (middle), and PAD4 levels (right) in PMA-stimulated CasRx-02-transfected dHL-60 cells in which miR-146a was silenced by creating sh-miR-146a-transfected stable transfectants. Values are mean ± SEM. All results in this figure were representative of at least 2 independent experiments with similar findings. * *p* < 0.05, ** *p* < 0.01, *** *p* < 0.001. DR: direct repeat, HA: human influenza hemagglutinin amino acids 98–106. NLS: nuclear localization sequence

A 2.7 kb stuffer was removed from pAll-EF1a-CasRx (Fig. 5a) for cloning crRNAs targeting SNHG16. Sorted GFP-positive CasRX-01 to -09- and CasRX-NC-transfected 293 T cells were examined for SNHG16 expression (Fig. 5b), with the highest silencing efficacy in CasRX-02-transfected cells. Furthermore, in CasRX-01-, -02-, -03-, -04- and -05-transfected cells with observed knockdown efficacy in SNHG16 expression varying from 27.9 to 70.2%, reciprocally up-regulated miR-146a expression was found in CasRX-01-, -02-, -03 and -04-, but not in -05-transfected cells (Fig. 5c, for SNHG16, CasRX-NC versus CasRX-01, 100.0 ± 4.5% versus 49.7 ± 6.3%, *p* < 0.001, CasRX-02, versus 29.8 ± 1.2%, *p* < 0.001, CasRX-03, versus 33.7 ± 0.4%, *p* < 0.001, CasRX-04, versus 53.4 ± 3.0%, *p* < 0.001, CasRX-05, versus 72.1 ± 9.0%, *p* = 0.041, for miR-146a, CasRX-NC versus CasRX-01, 100.0 ± 3.8% versus 151.5 ± 18.3%, *p* = 0.009, CasRX-02, versus 223.2 ± 24.0%, *p* < 0.001, CasRX-03, versus 184.7 ± 19.4% *p* = 0.002, CasRX-04 versus 139.6 ± 12.1% *p* = 0.033, CasRX-05, versus 111.6 ± 5.9%, *p* = 0.141). Sorted CasRX-02-transfected HL-60 cells were examined for SNHG16 and miR-146a expression (Fig. 5d, CasRX-NC versus CasRX-02, for SNHG16, 100.0 ± 1.5 versus 19.4 ± 1.6%, *p* < 0.001, for miR-146a, 100.0 ± 2.6 versus 357.6 ± 27.6%, *p* < 0.001). After PMA stimulation, decreased percentages of NETs morphology, and levels of CitH3 and PAD4 were found in sorted CasRX-02-transfected dHL-60 cells (Fig. 5e, CasRX-02 versus CasRX-NC, for NETs, 21.0 ± 5.1 versus 40.3 ± 3.8%, *p* = 0.038, for CitH3, 1.42 ± 0.12 versus 2.52 ± 0.06 ng/mL, *p* = 0.014, for PAD4, 2.98 ± 0.02 versus 5.27 ± 0.26 ng/mL, *p* = 0.012).

Furthermore, we knocked down miR-146a expression in CasRX-02-transfected HL-60 cells to demonstrate that up-regulated miR-146a levels is involved in the action mechanisms of reduced NETosis in PMA-stimulated SNHG16-silenced dHL-60 cells. Stable transfectants were created in which miR-146a was silenced in sorted CRISPR-CasRX-02-transfected HL-60 cells. After stimulation of DMSO-induced differentiated status by PMA, higher percentages of NETs morphology and levels of CitH3 and PAD4, were found in sh-miR-146a-transfected than sh-luciferase-transfected dHL-60 transfectants (Fig. 5f, sh-miR-146a versus sh-luciferase, for NETs, 27.7 ± 2.0 versus 18.7 ± 1.5%, *p* = 0.023, for CitH3, 1.64 ± 0.03 versus 0.88 ± 0.03 ng/mL, *p* = 0.002, for PAD4, 3.49 ± 0.05 versus 2.28 ± 0.31 ng/mL, *p* = 0.032).

Collectively, these findings indicated that overexpressing miR-146a or silencing SNHG16 to increase miR-146a expression can reduce NETs formation in human promyelocytic cells.

### Down-regulated miR-146a expression with increased NETs and apoptosis formation in mouse DAH lungs

**Fig. 6:**
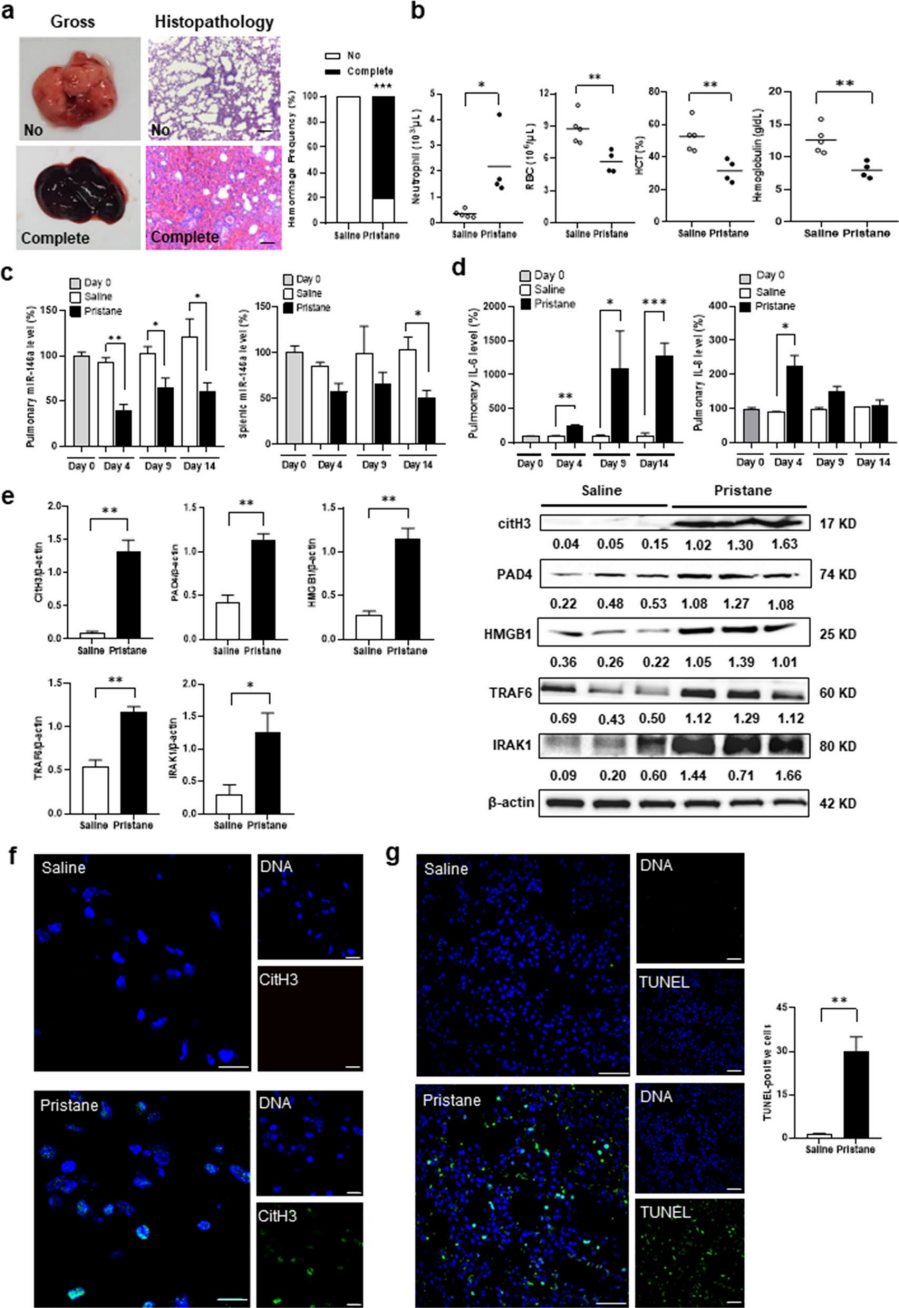
Down-regulated miR-146a expression with increased NETs and apoptosis formation in the mouse DAH lungs. **a** Right, representative gross and histopathological photographs in the mouse lungs with no and complete hemorrhage. Left, hemorrhage frequencies in saline- and pristane-injected mice. Scale bar = 100 µm, magnification × 100. **b** Neutrophil, RBC numbers, Hct and Hb levels in saline- and pristane-injected mice. **c** MiR-146a pulmonary (left) and splenic (right) levels on day 0, 4, 9 and 14 from saline- and pristane-injected mice. **d** IL-6 (left) and IL-8 (right) pulmonary levels on day 0, 4, 9 and 14 from saline- and pristane-injected mice. **e** Representative immunoblot assay (right) with signal intensity quantitation (left) of pulmonary CitH3, PAD4, HMGB1, TRAF6, IRAK1 and β-actin expression from saline- and pristane-injected mice. **f** Representative CitH3 IF staining (green) in lung tissues from saline- and pristane-injected mice. Cell nuclei counterstained with Hoechst 33,258 (blue). Scale bar = 12.5 µm, magnification × 800. **g** Left, representative TUNEL IF staining (green) in lung tissues from saline- and pristane-injected mice. Right, quantification of TUNEL-positive cell numbers in lung tissues. Cell nuclei counterstained with DAPI (blue). Scale bar = 25 µm, magnification, × 400. Values are mean ± SEM. Horizontal lines are mean values. Mouse numbers per group, 10 in **a**, 4 or 5 in **b**, 5 in **c**, **d**, 3 in **e**, 5 in **g**. All results in this figure were representative of 2 independent experiments with similar findings. * *p* < 0.05, ** *p* < 0.01, *** *p* < 0.001

DAH in a pristane-induced mouse model was demonstrated on day 14 with complete hemorrhage in 80% pristane-injected mice and 0% PBS-injected controls (Fig. 6a,  p < 0.001). There were higher neutrophil numbers, and lower RBC numbers, Hct and Hb levels in pristane-injected than control mice (Fig. 6b, for neutrophil, *p* = 0.018, for RBC, *p* = 0.008, for Hct, *p* = 0.007, for Hb, *p* = 0.006). Down-regulated pulmonary miR-146a levels were found in pristane-injected mice since day 4 (Fig. 6c, day 4, *p* = 0.003, day 9, *p* = 0.025, day 14, *p* = 0.046). Splenic miR-146a levels were also down-regulated in pristane-injected mice (day 14, *p* = 0.025). There were up-regulated pulmonary levels of IL-6 and IL-8 in pristane-injected mice (Fig. 6d, for IL-6, day 4, *p* = 0.001, day 9, *p* = 0.049, day 14, *p* < 0.001, for IL-8, day 4, *p* = 0.013). CitH3 and PAD4 levels in lung tissues were increased in pristane-injected mice (Fig. 6e, for CitH3, *p* = 0.002, for PAD4,* p* = 0.003), while distinct expression of CitH3 colocalized with DNAs, favoring NETs formation, was identified in pristane-injected but not control mice (Fig. 6f). Levels of HMGB1 and miR-146a target molecules IRAK1 and TRAF6, were increased in pristane-injected mice (Fig. 6e, for HMGB1, *p* = 0.001, for TRAF6, *p* = 0.004, for IRAK1, *p* = 0.017). Furthermore, TUNEL-positive apoptotic cells were found in pristane-injected but barely detected in PBS-treated lung tissues (Fig. 6g, 30.2 ± 4.8 versus 1.2 ± 0.4, *p* < 0.001).

### Down-regulated miR-146a expression with increased NETs formation in mouse neutrophils

**Fig. 7:**
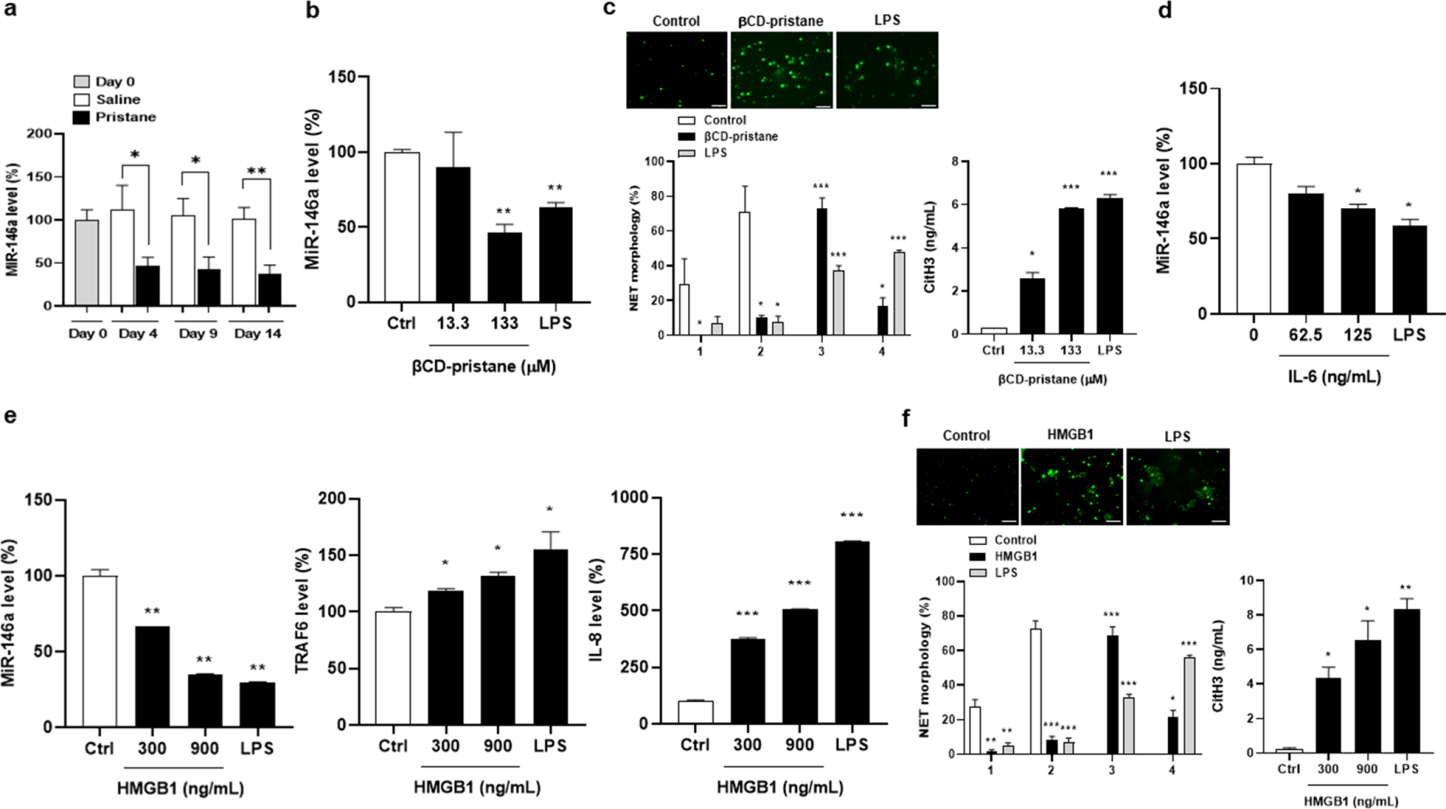
Down-regulated miR-146a expression with increased NETs formation in mouse neutrophils. **a** MiR-146a levels in thioglycolate-induced neutrophils purified from saline- and pristane-injected mice on day 0, 4, 9 and 14. **b**, MIR-146a levels in neutrophils stimulated with variable concentrations of βCD-pristane or 1 μg/mL LPS. **c** Left upper, representative photographs from neutrophil under 133 μM βCD-pristane or 1 μg/mL LPS stimulation. Scale bar = 30 µm, magnification × 400. Left lower, quantification of NETs morphology. Right, CitH3 levels in supernatants. **d** MiR-146a expression in neutrophils stimulated with various concentrations of IL-6 or 1 μg/mL LPS. **e** MIR-146a (left), TRAF6 (middle) and IL-8 (right) levels in neutrophils stimulated with variable concentrations of HMGB1 or 5 μg/mL LPS. **f** Left upper, representative photographs from neutrophil under 900 ng/mL HMGB1 or 5 μg/mL LPS stimulation. Scale bar = 30 µm, magnification × 400. Left lower, quantification of NETs morphology. Right, CitH3 levels in supernatants. Values are mean ± SEM. 5 mice per group in **a**. All results in Fig. 7 were representative of 2 independent experiments with similar findings. * *p* < 0.05, ** *p* < 0.01, *** *p* < 0.001

Pristane-complexed β-CD has been demonstrated to induce NETs formation in mouse neutrophils [28]. We further examined whether pristane-induced NETosis was through down-regulating miR-146a expression in mouse neutrophils. Before and after receiving pristane injection on day 0, 4, 9 and 14, neutrophils were isolated from thioglycolate-injected mice. There was down-regulated miR-146a expression since day 4 (Fig. 7a, day 4, *p* = 0.034, day 9, *p* = 0.047, day 14, *p* = 0.008). Neutrophils from day 0 naïve mice were cultured under the stimulation of βCD-pristane, IL-6, HMGB1 or LPS as a PC. In the presence of 13.3 μM, 133 μM βCD-pristane or 1 μg/mL LPS, there were down-regulated miR-146a expression (Fig. 7b), and increased diffused/spread NETs percentages with higher CitH3 production levels (Fig. 7c). Furthermore, miR-146a levels were down-regulated under the stimulation of 62.5 ng/mL, 125 ng/mL IL-6 or 1 μg/mL LPS (Fig. 7d). Notably, IL-8 is a well-known NETosis inducer as demonstrated by earlier experiments with the presence of IL-8 in mouse or human neutrophils cultures [29]. Under the stimulation of 300 ng/mL, 900 ng/mL HMGB1 or 5 μg/mL LPS, there were down-regulated miR-146a, up-regulated TRAF6 and IL-8 expression (Fig. 7e), and increased diffused/spread NETs percentages with higher CitH3 production levels (Fig. 7f).

### Reduced apoptosis and HMGB1 release in miR-146a-overexpressed MLE-12 cells

**Fig. 8:**
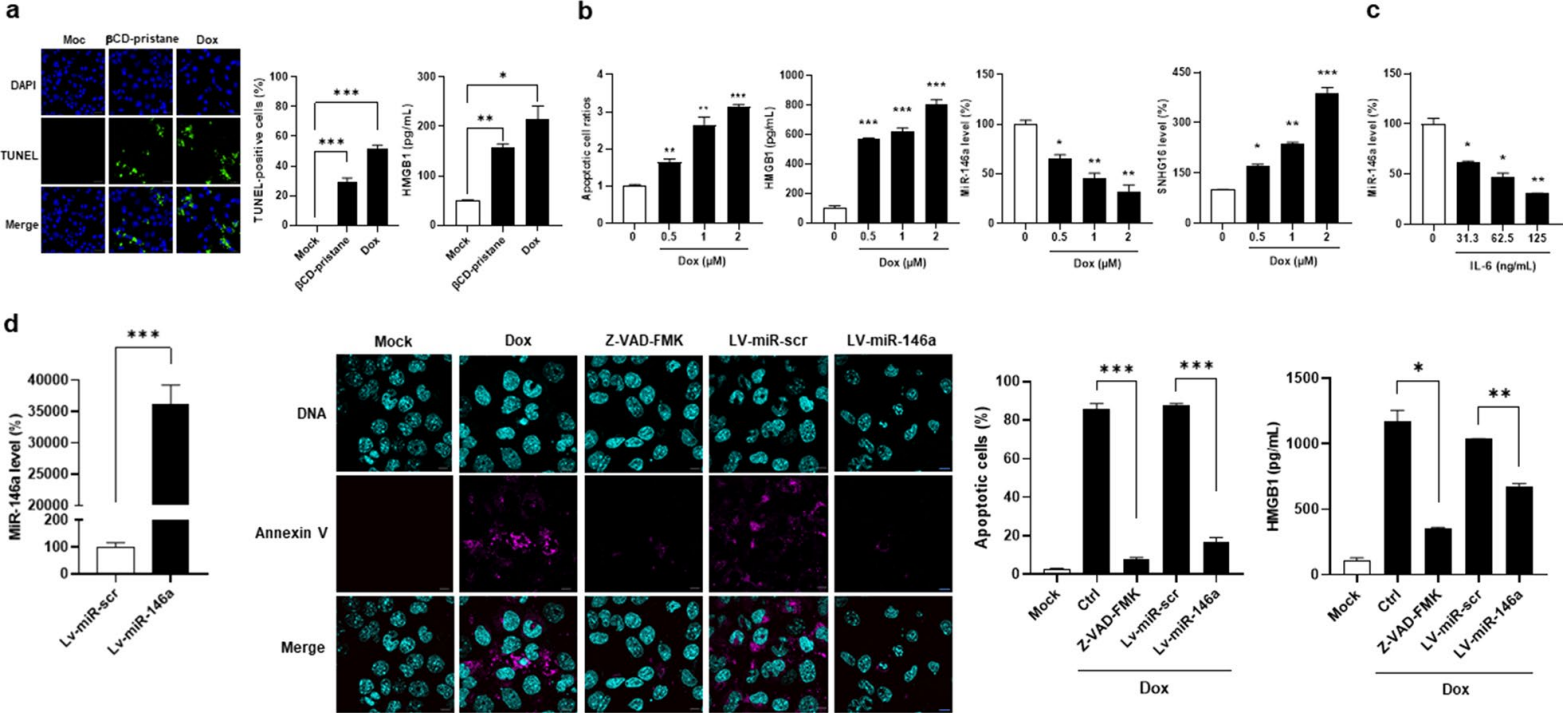
Reduced apoptosis and HMGB1 production in miR-146a-overexpressed alveolar cells. **a** Left, representative photographs of TUNEL IF staining (green) in MLE-12 cells stimulated with 400 μM βCD-pristane or 1 μM Dox. Cell nuclei counterstained with DAPI (blue). Scale bar = 30 µm, magnification × 600. Middle, quantification of TUNEL-positive cell percentages. Right, HMGB1 supernatant levels from stimulated MLE-12 cells. **b** Apoptotic cell ratios (left), HMGB1 supernatant levels (middle left), miR-146a (middle right) and SNHG16 (right) expression in MLE-12 cells stimulated with various concentrations of Dox. **c** MiR-146a expression in MLE-12 cells stimulated with various concentrations of IL-6. **d** Left, MiR-146a expression in LV-miR-146a-transfected MLE-12 cells. Middle left, representative photographs of annexin V IF staining (pink) in miR-scr- or miR-146a-overexpressed MLE-12 cells under 1 μM Dox stimulation. Cell nuclei counterstained with Hoechst 33258 (blue). Scale bar = 10 µm, magnification × 1000. Middle right, quantification of apoptotic cell percentages. Right, HMGB1 supernatant levels from miR-scr- or miR-146a-overexpressed MLE-12 cells under 1 μM Dox stimulation. Values are mean ± SEM. All results in this figure were representative of 3 independent experiments with similar findings. * *p* < 0.05, ** *p* < 0.01, *** *p* < 0.001

TLR4-expressed neutrophils have been shown to be activated by HMGB1, functioning as damage-associated molecular pattern (DAMP) molecule to induce NETs formation [30]. MiR-146a inhibits both of the Fas-mediated and p53-dependent apoptosis [8, 9], whereas βCD-pristane promotes the p53-dependent apoptosis [5]. In this study, we examined whether overexpressing miR-146a in alveolar cells could inhibit apoptosis to reduce HMGB1 release. There were increased apoptotic percentages and HMGB1 levels in MLE-12 cells culture, greater under Dox than under βCD-pristane stimulation (Fig. 8a, for apoptosis, mock versus βCD-pristane, 0 ± 0 versus 29.4 ± 5.6%, *p* < 0.001, or Dox, versus 52.1 ± 1.9%, *p* < 0.001, for HMGB1, mock versus βCD-pristane, 51.1 ± 0.4 versus 156.8 ± 7.9 pg/mL, *p* = 0.006, or Dox, versus 214.8 ± 27.0 pg/mL, *p* = 0.026). In addition, βCD-pristane-stimulated MLE-12 cells had a dose-dependent increase in apoptotic cell ratios, HMGB1 and IL-8 levels with down-regulated miR-146a and up-regulated TRAF6 and SNHG16 expression (Additional file 2: Fig. S2a to f).

In Fig. 8b, Dox-stimulated MLE-12 cells had a dose-dependent increase in apoptotic cell ratios and HMGB1 production levels with down-regulated miR-146a and up-regulated SNHG16 expression. Notably, miR-146a levels in MLE-12 cells were down-regulated under the stimulation of IL-6 in a dose-dependent manner (Fig. 8c). MiR-146a-transfected MLE-12 cells had increased miR-146a levels (Fig. 8d, miR-146a versus miR-scr, 36,157.2 ± 3,090.0 versus 100.0 ± 16.2%, *p* < 0.001). After Dox stimulation, lower apoptotic percentages and decreased HMGB1 levels were found in miR-146a-overexpressed MLE-12 cells (miR-146a versus miR-scr, for apoptosis, 17.1 ± 2.0 versus 87.9 ± 0.6%, *p* < 0.001, for HMGB1, 678.4 ± 21.9 versus 1,036.0 ± 2.7 pg/mL, *p* = 0.004).

### DAH suppressed by intra-pulmonary miR-146a delivery through reducing NETs and apoptosis formation

**Fig. 9:**
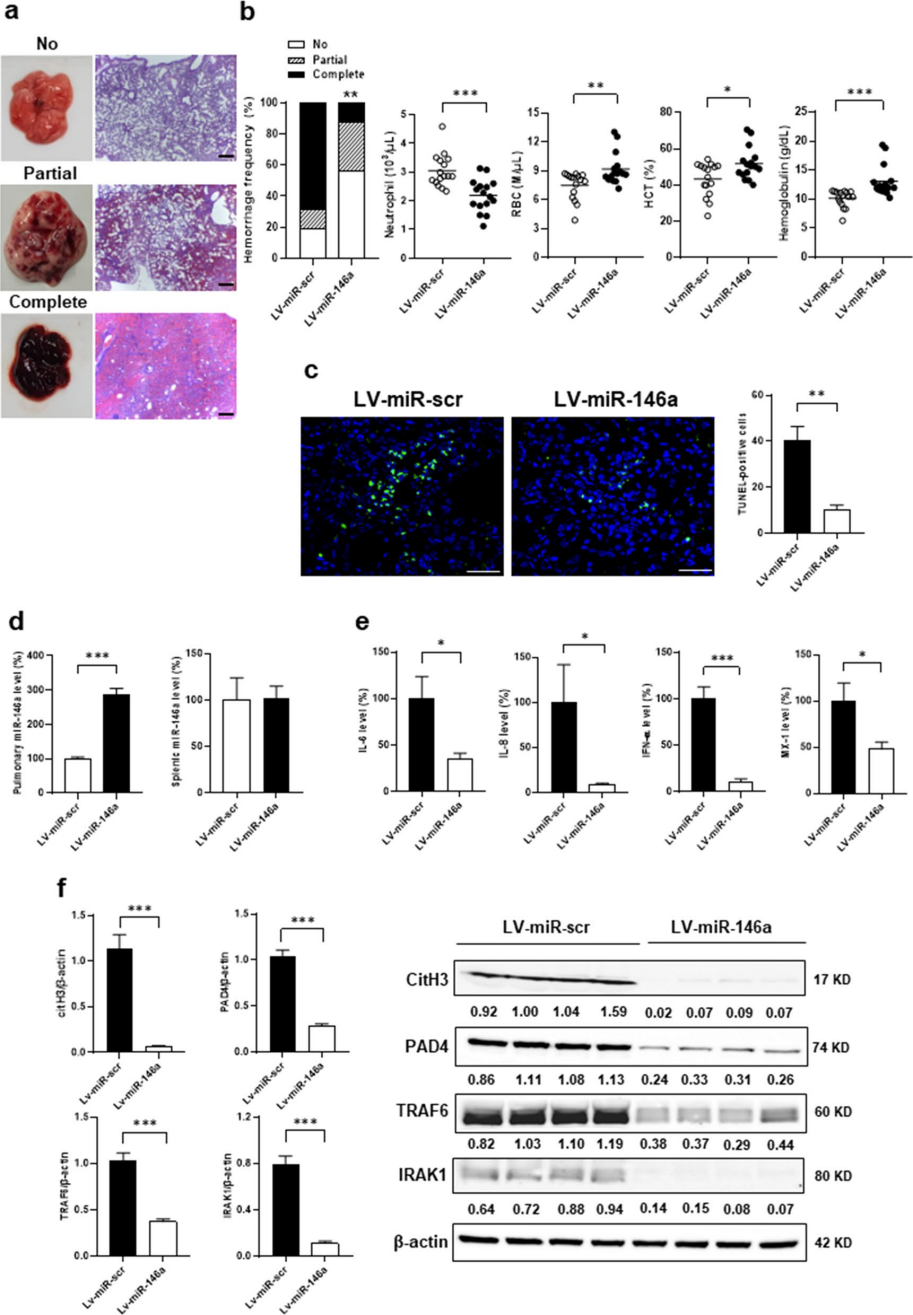
DAH suppressed by intra-pulmonary miR-146a delivery through reducing NETs and apoptosis formation. **a** Left, representative gross and histopathological photographs in the lungs with no, partial and complete hemorrhage. Right, hemorrhagic frequencies of LV-miR-scr- and LV-miR-146a-treated mice. Scale bar = 100 µm, magnification, × 100. **b** Neutrophils, RBC numbers, Hct and Hb levels in LV-miR-scr- and LV-miR-146a-treated mice. **c** Left, representative TUNNEL IF staining (green) in lung tissues from LV-miR-scr- and LV-miR-146a-treated mice. Scale bar = 20 µm, magnification, × 400. Right, quantification of TUNEL-positive cell numbers in lung tissues. **d** MiR-146a levels in lung and spleen tissues from LV-miR-scr- and LV-miR-146a-treated mice. **e** Levels of IL-6, IL-8, IFN-α and MX-1 in lung tissues from LV-miR-scr- and LV-miR-146a-treated mice. **f** Representative immunoblot assay (right) with signal intensity quantitation (left) of pulmonary CitH3, PAD4, TRAF6, IRAK1 and β-actin levels from saline- and pristane-injected mice. Values are mean ± SEM. Horizontal lines are mean values. Mouse numbers per group, 16 in **a**, **b**, 5 in **c**, 8 in **d**, 8 in **e**, 4 in **f**. All results in this figure were representative of 2 independent experiments with similar findings. * *p* < 0.05, ** *p* < 0.01, *** *p* < 0.001

C57BL/6 mice receiving intra-tracheal LV-miR-146a delivery had lower complete hemorrhage frequencies than LV-miR-scr-treated controls (Fig. 9a, 12.5% versus 68.8%, *p* = 0.003). There were lower neutrophil numbers, and higher RBC numbers, Hct and Hb levels in LV-miR-146a-treated mice (Fig. 9b, for neutrophil, *p* < 0.001, for RBC, *p* = 0.004, for Hct, *p* = 0.012, for Hb, *p* < 0.001). Lower pulmonary TUNEL-positive cell numbers were found in LV-miR-146a-treated mice (Fig. 9c, 10.2 ± 2.1 versus 40.6 ± 5.8, *p* = 0.001). LV-miR-146a-treated mice had higher pulmonary miR-146a levels (Fig. 9d, miR-146a versus miR-scr, 287.0 ± 17.9 versus 100.0 ± 5.2%, *p* < 0.001), while no differences were found in splenic expression between two treatment groups (miR-146a versus miR-scr, 102.4 ± 12.8 versus 100.0 ± 23.9%, *p* = 0.933). There were decreased pulmonary levels of IL-6 and IL-8 in LV-miR-146a-treated mice (Fig. 9e, for IL-6, *p* = 0.020, for IL-8, *p* = 0.047). The pristane-induced mouse model is driven by a strong type I IFN response which can be targeted by miR-146a [5, 9]. LV-miR-146a-treated mice had lower pulmonary levels of IFN-α and MX-1 (Fig. 9e, for IFN-α, *p* < 0.001, for MX-1, *p* = 0.027). There was reduced expression of CitH3, PAD4, TRAF6 and IRAK-1 in LV-miR-146a-treated lung tissues (Fig. 9f,  p < 0.001 in all).

**Fig. 10:**
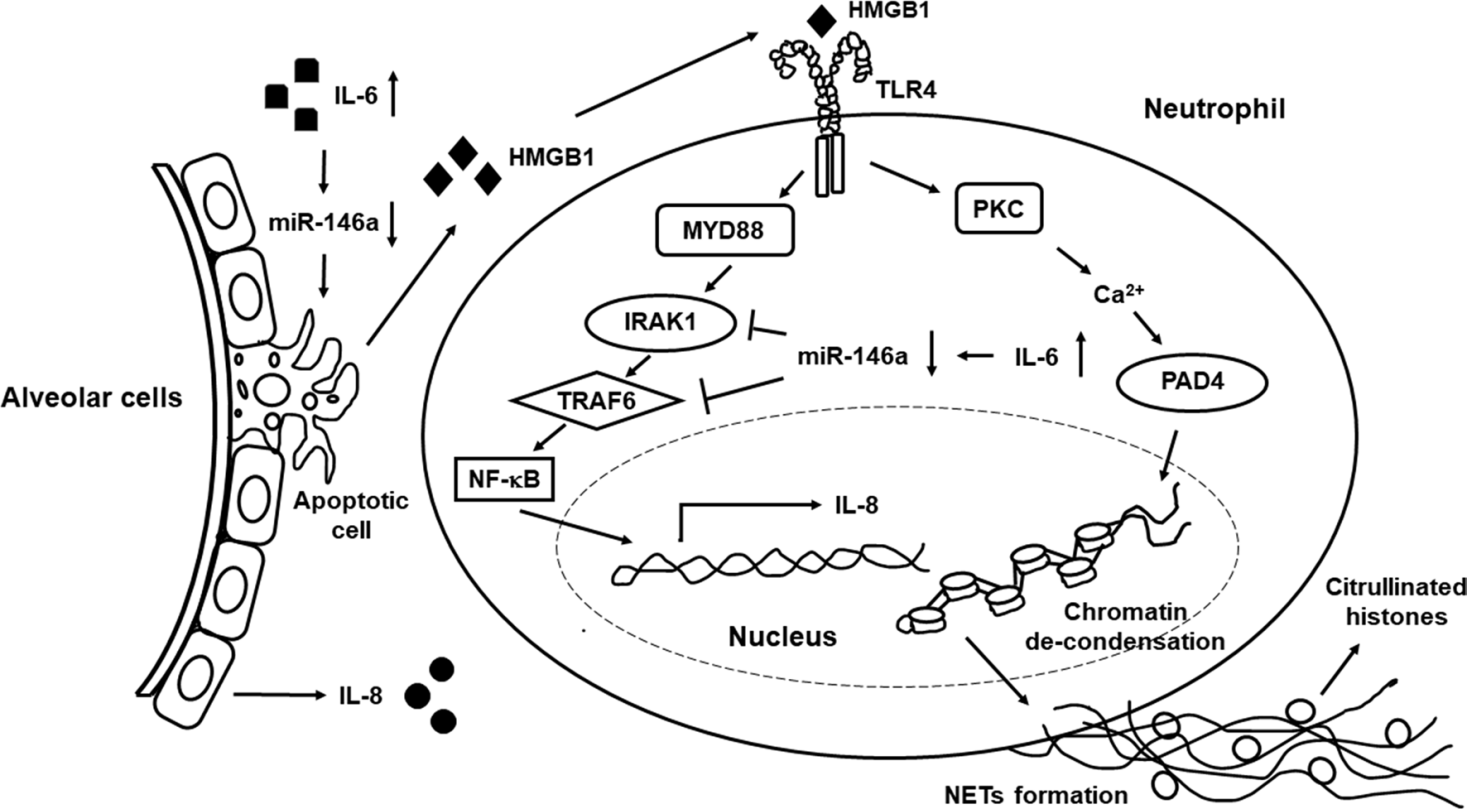
A schematic representation summarizing the experimental results. Under increased pulmonary IL-6 expression, down-regulated miR-146a expression in alveolar cells can induce cell apoptosis with the release of HMGB1, functioning as DAMP molecule to engage TLR4-expressed neutrophils, followed by activated PKC to mobilize intracellular Ca^2 +^ and promote PAD4 activation, leading to NETs formation. Furthermore, through down-regulating miR-146a levels by IL-6 stimulation, up-regulated expression of the target molecule TRAF6 in alveolar cells and neutrophils can enhance the secretion of IL-8, a well-known inducer of NETosis

Figure 10 is a schematic representation summarizing the above experimental results. Under increased pulmonary IL-6 expression, down-regulated miR-146a levels in alveolar cells can induce cell apoptosis with the release of HMGB1, functioning as DAMP molecule to engage TLR4-expressed neutrophils, followed by activated protein kinase C (PKC) to mobilize intracellular Ca^2 +^ and promote PAD4 activation, leading to NETs formation. Furthermore, through down-regulating miR-146a levels by IL-6 stimulation, up-regulated expression of the target molecule TRAF6 in alveolar cells and neutrophils can enhance the secretion of IL-8, a well-known inducer of NETosis.

### Up-regulated renal miR-146a expression in LN patients and a mouse model

Renal involvement is a common disease morbidity in SLE [3]. LN patients had lower miR-146a levels in PBMCs than those without renal involvement or HCs (Additional file 3: Fig. S3a, left, LN versus Nil, 37.6 ± 7.2 versus 79.1 ± 15.2%, *p* = 0.003, or HCs, versus 100.0 ± 12.4%, *p* < 0.001), and there was a negative correlation between miR-146a levels and daily proteinuria amounts in SLE (Additional file 3: Fig. S3a, right, r = − 0.364, *p* = 0.004). Nevertheless, miR-146a levels in USCs were higher in LN patients than no renal involvement or HCs (Additional file 3: Fig. S3b, LN versus Nil, 570.6 ± 204.7 versus 138.3 ± 82.3%, *p* = 0.002, or HCs, versus 100.0 ± 26.9%, *p* = 0.031).

At month 6 after pristane injection, there were hyper-cellularity and mesangial expansion in renal glomeruli (Additional file 3: Fig. S3c). At month 5 and 6, increased proteinuria levels were found in pristane-injected mice (100% incidence, Additional file 3: Fig. S3c, left, month 5, *p* = 0.028, month 6, *p* = 0.002). In addition, higher anti-dsDNA titers were noted at month 5 and 6 (100% incidence, Additional file 3: Fig. S3c, right, month 5, *p* = 0.006, month 6, *p* < 0.001). Upon sacrifice, the kidneys were removed for examining miR-146a expression before and after pristane injection and at month 0, 1, 3, 5 and 6 (Additional file 3: Fig. S3d). During the earlier GN development stage at month 1, there were down-regulated miR-146a expression, similar to the results of pristane-injected lung tissues on day 14, and up-regulated levels were found at month 5 and 6 (pristane versus saline, for month 1, 65.9 ± 10.3 versus 99.3 ± 9.7%, *p* = 0.041, for month 3, 106.3 ± 5.6 versus 98.7 ± 8.1%, *p* = 0.226, for month 5, 220.6 ± 39.5 versus 98.1 ± 17.7%, *p* = 0.018, for month 6, 309.6 ± 11.4 versus 97.6 ± 12.4%, *p* < 0.001).

In addition, at month 5 and 6 after pristane injection, arthritis (Additional file 4: Fig. S4a) was found in two out of 12 mice (17% incidence, Additional file 4: Fig. S4b), but not in PBS-injected mice. There were no differences in renal miR-146a levels between mice with and without arthritis (with versus without, 291.9 ± 17.2 versus 259.8 ± 28.3%, *p* = 0.637).

From the results of miR-146a levels in PBMCs from LN patients and renal miR-146a expression in the earlier GN development stage of pristane-induced mice, down-regulated miR-146a expression might contribute to a part of the LN pathogenesis. Nevertheless, in contrast to down-regulated miR-146a levels in the DAH-related pulmonary specimens, our experiments demonstrated up-regulated expression in the established GN-associated renal samples. Indeed, the expression levels of miRNAs are highly responsive to the distinct kinetics of cytokine profiles in different target organs of SLE patients [26]. Based on above findings, besides a therapeutic potential of earlier intra-renal miR-146a delivery, medications modifying the specific intra-renal cytokine milieu might provide the beneficial efficacy in LN patients.

## Discussion

In this study, down-regulated and up-regulated miR-146a levels were found in PBMCs and USCs from LN, respectively. Down-regulated miR-146a levels in PBMCs has been demonstrated in SLE, negatively associated with increased IL-6/IL-8 and TRAF6 expression, particularly in renal involvement; however, miR-146a levels were up-regulated in USCs and glomerular tissues from LN [9, 31]. Notably, there was predominant expression of Th1 cytokines, IFN-γ/IL-2, in USCs from LN [32]. Since cytokines can induce cellular miRNAs expression [9, 26], conflicting findings between miR-146a levels in PBMCs and USCs might reflect inconsistent circulating and renal cytokine profiles in LN. In our experiments, DAH lung tissues had higher IL-6 but lower miR-146a expression. MiR-146a levels in neutrophils or alveolar cells culture were down-regulated by the IL-6 stimulation. MiRNAs can be epigenetically regulated by IL-6, while miR-146a promoter has CpG island with putative STAT-1/NF-κB binding sites [31]. Further studies are needed to elucidate whether down-regulated miR-146a expression in DAH lung tissues is caused by IL-6-induced DNAs methylation.

MiR-146a −/− mice receiving the injection of LPS, a TLR4 agonist, had increased NETs formation [33], whereas TLR4 −/− mouse neutrophils were associated with reduced NETosis [34]. Oxidized low density lipoprotein could induce NETs formation in neutrophils via TLRs activation with IRAK/PKC/MAK pathways as signaling mediators [35]. These findings suggest that lower miR-146a levels enhance NETs formation through regulating the TLRs signaling. In this study, there was down-regulated miR-146a expression in dHL-60 cells during NETosis, while miR-146a-overexpressed dHL-60 cells had reduced NETs formation. Lower miR-146a levels in PBNs from DAH patients caused greater NETs formation with higher amounts of ICs formation/deposition, leading to severer activity than other patients. SLE-associated or pristane-induced DAH lung tissues had enhanced NETosis with down-regulated miR-146a and up-regulated TRAF6 expression, while intra-pulmonary miR-146a delivery could lessen TRAF6 levels to reduce NETs formation. Our experimental data implicated that pulmonary NETosis in DAH is regulated by miR-146a through targeting the expression of TLRs pathways-related molecule TRAF6.

IL-8 is secreted by cells expressing TLRs in response to inflammatory stimuli [36]. Although neutrophils migrate when sensing the IL-8 gradient, higher concentrations can saturate the receptors and prohibit their chemotaxis, followed by NETs formation [37]. Increased NETs formation and IL-8 levels were identified in bronchoalveolar lavage fluid from pneumonia patients [38], while reduced miR-146a with higher IL-8 expression was demonstrated in biopsied asthmatic bronchial cells [39]. Despite no identification of MREs within IL-8 mRNA by existing algorithms, miR-146a can negatively regulate IL-8 production at the translational level [40]. In our experiments, the DAH lungs had down-regulated miR-146a, up-regulated TRAF6 and increased IL-8 expression with enhanced NETosis, while intra-pulmonary miR-146a delivery could suppress TRAF6 and IL-8 expression with reduced NETs formation. Besides, stimulated neutrophils and alveolar cells had down-regulated miR-146a, and up-regulated TRAF6 with increased IL-8 expression. These results indicated that, in DAH, miR-146a can regulate pulmonary NETs formation by targeting TRAF6 to modulate IL-8 expression.

Pulmonary NETosis with colocalized CitH3 and DNAs, has been demonstrated in the pristane-injected lungs, while DNase-1 inhalation to clear NETs could suppress mouse DAH [28]. Reduced DAH severity was shown in mice with myeloid-specific PAD4 deletion, displaying a protective role with the loss of PAD4-dependent NETosis [6]. We identified NETs formation with down-regulated miR-146a expression in human and mouse DAH lung tissues, while intra-pulmonary miR-146a delivery could suppress DAH through reducing NETosis. Collectively, these findings suggested the involvement of miR-146a-regulated NETs formation and the potential of anti-NETs therapy in DAH patients.

HMGB1 is released from activated or damaged cells, serving as DAMP molecule to interact with TLRs-expressed cells and participate in autoimmune responses [41]. In particular, apoptotic cells are an important source of HMGB1 [42]. Circulating HMGB1 levels were positively correlated with disease activity in SLE [41]. Furthermore, pristane-injected mice released HMGB1 from apoptotic cells, activating neutrophils through the TLRs signaling to form NETs [43]. Incubation of bone marrow-derived mouse neutrophils with HMGB1 could induce NETs formation [44], while our experiments demonstrated HMGB1-induced NETosis in cultured thioglycolate-activated peritoneal mouse neutrophils. In this study, DAH lung tissues had down-regulated miR-146a levels with increased apoptosis and HMGB1 expression. Stimulated alveolar cells had down-regulate miR-146a expression with increased apoptosis and HMGB1 release, while miR-146a-overexpressed cells had reduced apoptosis and HMGB1 production. Our experimental results demonstrated that increased apoptosis by down-regulated miR-146a expression in alveolar cells could enhance the HMGB1 release, engaging with TLR4-expressed neutrophils to induce pulmonary NETosis.

Although RNA interference attacks cytosolic RNAs for degradation, it is ineffective in targeting intra-nuclear lncRNAs [45]. Small interfering RNA or shRNA can induce TLRs-mediated innate immune responses, and has the potential of large-scale off-target cleavage. CRISPR/Cas9 editing introduces irreversible DNA cleavage associated with a higher risk of off-target effects. CRISPR type VI nucleases have been discovered as site-specific RNA-guided, RNA-targeting effectors including Cas13a, b, c and d subtypes [21, 46]. Cas13d has higher silencing efficiency and specificity than other Cas13 subtypes-mediated RNA knockdown or CRISPR/Cas9-conducted DNA cleavage [46, 47]. In this study, we created an all-in-one CRISPR-Cas13d vector to target SNHG16 by screening guide RNA sequences. Significant efficacy was identified in crRNA-02 sequence silencing more than 80% expression with reduced NETosis in PMA-stimulated SNHG16-silenced dHL-60 cells.

Although no anti-NETs therapeutics have been approved, there are effective compounds like PAD4 inhibitors [48]. In this study, less NETs morphology and CitH3/PAD4 production were observed in PMA-stimulated dHL-60 cells with the presence of Cl-amidine, a PADs inhibitor. Cl-amidine injection protected MRL/lpr lupus mice from NETs-mediated injury [49], while injecting miR-155 antagomirs to inhibit the induction of PAD4 could suppress mouse DAH [13]. Nevertheless, there was impaired PAD4-related physiological function by systemic administration of its blockers [49, 50]. In SLE, there are variable pathogenic effects of NETs in different organs [4, 49], requiring focus on the targets when applying anti-NET therapeutics. By intra-tracheal delivery of LV-miR-146a, higher pulmonary levels were observed without differences in splenic expression, suggesting no extra-pulmonary leakage of infused vectors in our experiments. Indeed, intra-pulmonary delivery of anti-NETs compounds can minimize the adverse effects outside the lungs in DAH patients.

## Conclusions

Our results demonstrate firstly down-regulated pulmonary miR-146a levels with increased TRAF6 and IL-8 expression and NETs and apoptosis formation in autoimmune-mediated DAH, and implicate a therapeutic potential of intra-pulmonary miR-146a delivery in such patients.

## Supplementary Information

**Additional file 1: Fig. S1.** pLKO.1-sh-miR-146a #1 and miR-146a targeting efficacy in LV-sh-miR-146a #1, #2 or #3-transfected 293 T cells and LV-sh-miR-146a #1-transfected HL-60 stable transfectants. **a** Map of pLKO.1-sh-miR-146a #1 with a 1.9 kb stuffer removed by *Age*I and *Eco*RI cutting, a total of 7,518 bp in length. **b** MiR-146a targeting efficacy in LV-sh-miR-146a #1, #2 or #3-transduced 293 T cells. **c** MiR-146a targeting efficacy in LV-sh-miR-146a #1-transfected HL-60 stable transfectants. The expression levels of LV-sh-luciferase-transfected controls were determined as 100%. All of the in vitro results in Fig. S1 were representative of two independent experiments with similar findings. hPGK: human posphoglycerate kinase, Psi: RNA packaging signal, RRE: Rev response element, WPRE: woodchuck hepatitis virus posttranscriptional regulatory element

**Additional file 2: Fig. S2.** Biological responses in βCD-pristane-stimulated mouse alveolar cells. **a** Apoptotic cell ratios. **b** HMGB1 supernatant concentrations. **c** IL-8 expression levels. **d** MiR-146a expression levels. **e** TRAF6 expression levels. **f** SNHG16 expression levels. Values are mean ± SEM. Results in Fig. S2 were representative of 3 independent experiments with similar findings. * *p* < 0.05, ** *p* < 0.01, *** *p* < 0.001.

**Additional file 3: Fig. S3.** Up-regulated renal miR-146a expression in LN patients and mice. **a** Left, miR-146a levels in PBMCs from HCs, LN and Nil patients. Right, A negative correlation between miR-146a levels in PBMCs and daily proteinuria amounts from SLE patients. **b** MiR-146a levels in USCs from HCs, LN and Nil patients. **c** Left, PAS staining of renal glomeruli at month 6 after saline or pristane injection. Scale bar = 10 µm, magnification, × 400. Kinetic measurement of proteinuria levels in mice at month 0, 1, 3, 5 and 6. Right, kinetic measurement of anti-dsDNA titers at month 0, 1, 3, 5 and 6. **d** Renal miR-146a expression in mice after pristane induction at month 0, 1, 3, 5 and 6. Values are mean ± SEM. Horizontal lines are mean values. Patient numbers, **a** 40 for LN, 20 for Nil, **b** 15 for LN or Nil. Mouse numbers per group, 6 in **c** and **d**. Results of **c** and **d** in Fig. S3 were combined data of 2 independent experiments, 6 mice per each time point. * *p* < 0.05, ** *p* < 0.01, *** *p* < 0.001.

**Additional file 4: Fig. S4.** Arthritis in mice after pristane injection. **a** Left, no swollen joints in a Balb/C mouse after PBS injection at month 6. Right, representative swollen hind paw joints (white arrow) in a Balb/C mouse after pristane induction at month 6. **b** Incidence of arthritis in Balb/C mice after PBS or pristane injection at month 5 and 6. Mouse numbers per group, 12 in **b**. Results of **b** in Fig. S4 were combined data of 2 independent experiments with 6 mice per group.

## Data Availability

The datasets used and/or analyzed in the current study are available from the corresponding author upon reasonable request.

## Abbreviations

7AAD: 7-Amino-actinomycin D
ACR: American College of Rheumatology
βCD: β Cyclodextrin
cDNA: Complementary DNA
CitH3: Citrullinated Histone 3
Cl-amidine: Chloramidine
CRISPR: Clustered regularly interspaced short palindromic repeat
DAMP: Damage-associated molecular pattern
DAH: Diffuse alveolar hemorrhage
DMSO: Dimethyl sulfoxide
dHL-60: Differentiated HL-60
Dox: Doxorubicin
ELISA: Enzyme-linked immunosorbent assay
GN: Glomerulonephritis
HC: Healthy control
Hb: Hemoglobulin, Hct: Hematocrit
H&E: Hematoxylin and eosin
HMGB1: High-mobility group box 1
IC: Immune complex
IFN: Interferon
ISG: IFN-inducible gene
LncRNA: Long noncoding RNA
LN: Lupus nephritis
LPS: Lipopolysaccharide
LV: Lentivirus
MiRNA: MicroRNA
MRE: MiRNA recognition element
NC: Negative control
NET: Neutrophil extracellular trap
PAD4: Protein arginine deiminase 4
PAS: Periodic acid-Schiff
PBN: Peripheral blood neutrophil
PBS: Phosphate-buffered saline
PC: Positive control
PKC: Protein kinase C
PMA: Phorbol 12-myristate 13-acetate
PTX: Pneumothorax
qRT-PCR: Quantitative real time polymerase chain reaction
RBC: Red blood cells
RT: Room temperature
SEM: Standard error of mean
SLE: Systemic lupus erythematosus
TLR: Toll-like receptor
Tm: Melting temperature
TU: Transduction unit
TUNEL: Terminal deoxynucleotidyl transferase dUTP nick end labeling
USC: Urine sediment cell
