# Supplementary figures and images for: Down-regulated miR-146a expression with increased neutrophil extracellular traps and apoptosis formation in autoimmune-mediated diffuse alveolar hemorrhage
Source: J Biomed Sci. 2022 Aug 26;29:62. doi: 10.1186/s12929-022-00849-4 (PMC9413930; doi:10.1186/s12929-022-00849-4)

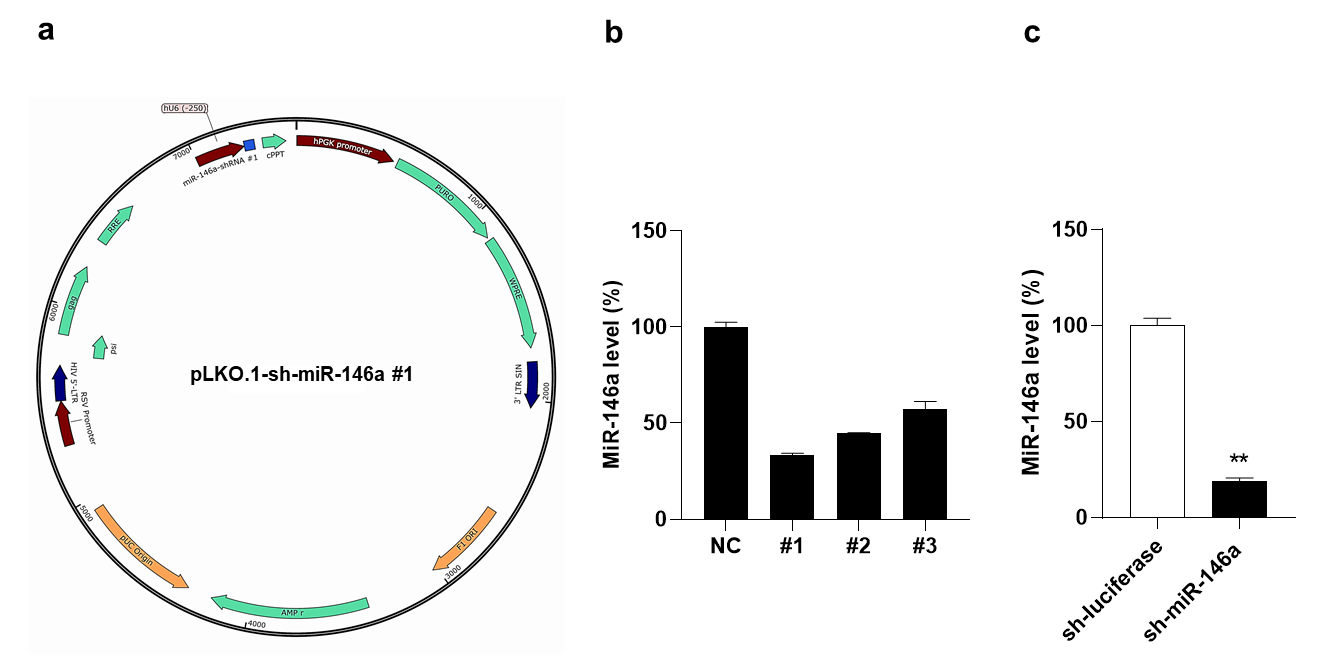

Supplement: Supplementary file 1 — Additional file 1: Fig. S1. pLKO.1-sh-miR-146a #1 and miR-146a targeting efficacy in LV-sh-miR-146a #1, #2 or #3-transfected 293 T cells and LV-sh-miR-146a #1-transfected HL-60 stable transfectants. a Map of pLKO.1-sh-miR-146a #1 with a 1.9 kb stuffer removed by AgeI and EcoRI cutting, a total of 7,518 bp in length. b MiR-146a targeting efficacy in LV-sh-miR-146a #1, #2 or #3-transduced 293 T cells. c MiR-146a targeting efficacy in LV-sh-miR-146a #1-transfected HL-60 stable transfectants. The expression levels of LV-sh-luciferase-transfected controls were determined as 100%. All of the in vitro results in Fig. S1 were representative of two independent experiments with similar findings. hPGK: human posphoglycerate kinase, Psi: RNA packaging signal, RRE: Rev response element, WPRE: woodchuck hepatitis virus posttranscriptional regulatory element [file 12929_2022_849_MOESM1_ESM.tif]

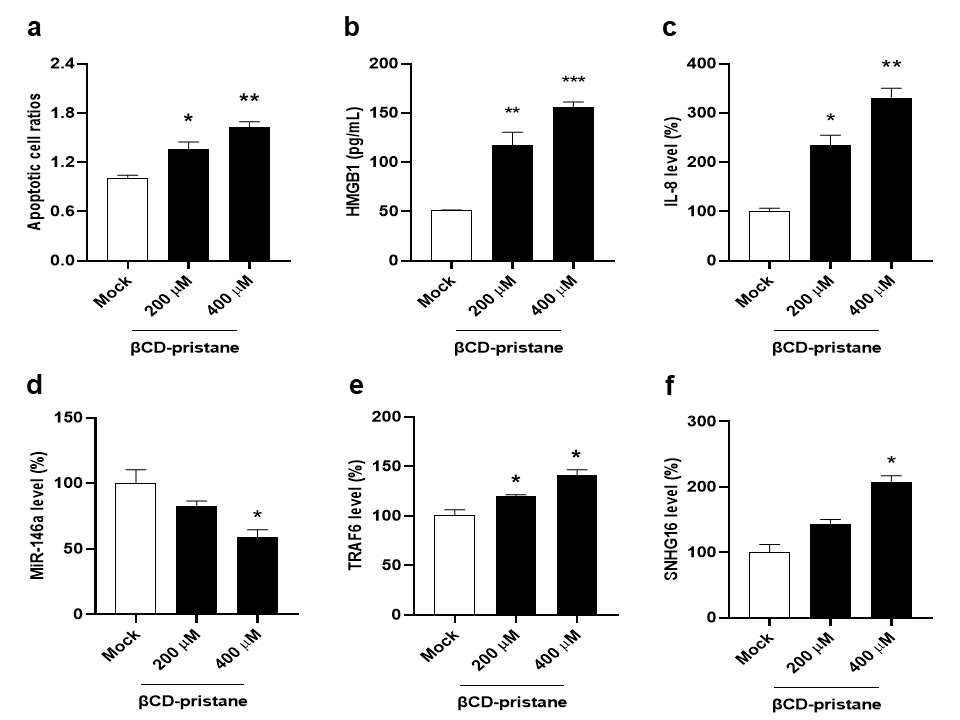

Supplement: Supplementary file 2 — Additional file 2: Fig. S2. Biological responses in βCD-pristane-stimulated mouse alveolar cells. a Apoptotic cell ratios. b HMGB1 supernatant concentrations. c IL-8 expression levels. d MiR-146a expression levels. e TRAF6 expression levels. f SNHG16 expression levels. Values are mean ± SEM. Results in Fig. S2 were representative of 3 independent experiments with similar findings. * p < 0.05, ** p < 0.01, *** p < 0.001. [file 12929_2022_849_MOESM2_ESM.tif]

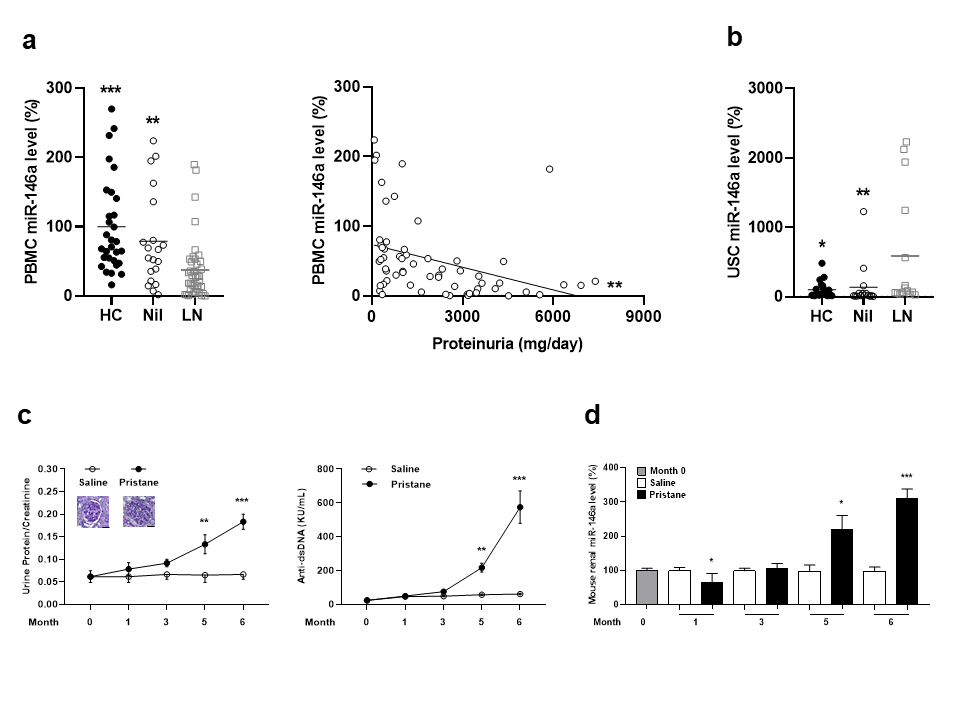

Supplement: Supplementary file 3 — Additional file 3: Fig. S3. Up-regulated renal miR-146a expression in LN patients and mice. a Left, miR-146a levels in PBMCs from HCs, LN and Nil patients. Right, A negative correlation between miR-146a levels in PBMCs and daily proteinuria amounts from SLE patients. b MiR-146a levels in USCs from HCs, LN and Nil patients. c Left, PAS staining of renal glomeruli at month 6 after saline or pristane injection. Scale bar = 10 µm, magnification, × 400. Kinetic measurement of proteinuria levels in mice at month 0, 1, 3, 5 and 6. Right, kinetic measurement of anti-dsDNA titers at month 0, 1, 3, 5 and 6. d Renal miR-146a expression in mice after pristane induction at month 0, 1, 3, 5 and 6. Values are mean ± SEM. Horizontal lines are mean values. Patient numbers, a 40 for LN, 20 for Nil, b 15 for LN or Nil. Mouse numbers per group, 6 in c and d. Results of c and d in Fig. S3 were combined data of 2 independent experiments, 6 mice per each time point. * p < 0.05, ** p < 0.01, *** p < 0.001. [file 12929_2022_849_MOESM3_ESM.tif]

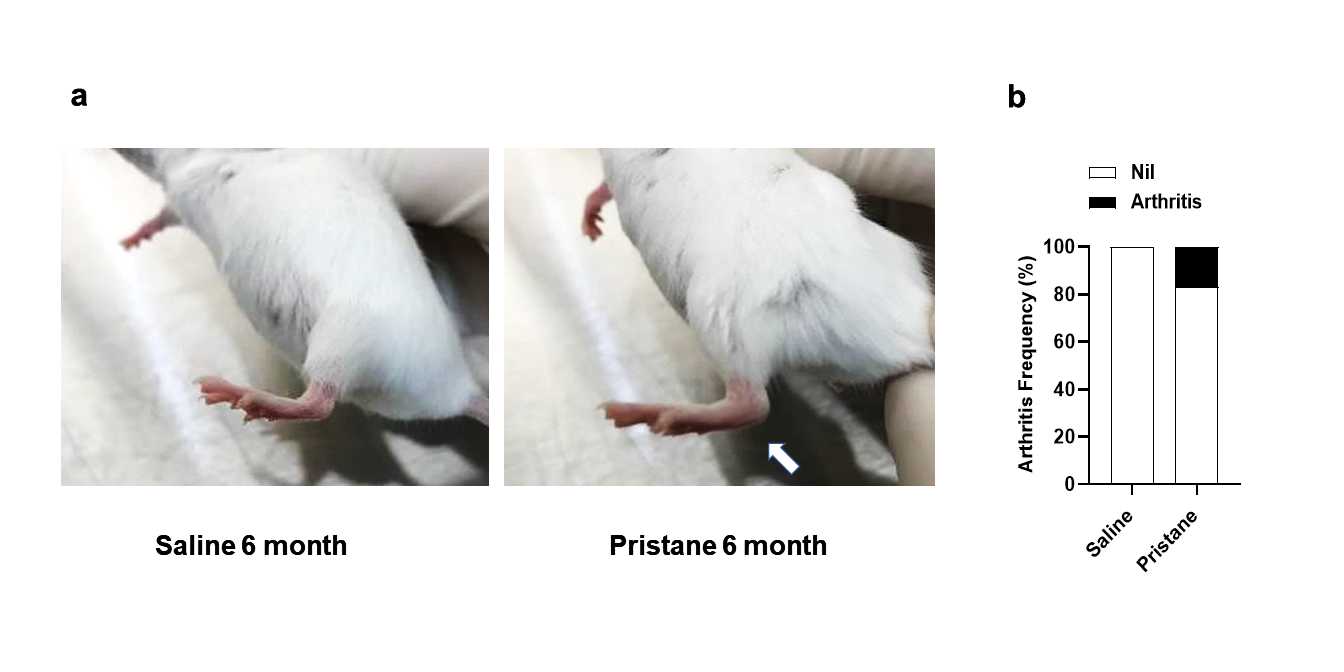

Supplement: Supplementary file 4 — Additional file 4: Fig. S4. Arthritis in mice after pristane injection. a Left, no swollen joints in a Balb/C mouse after PBS injection at month 6. Right, representative swollen hind paw joints (white arrow) in a Balb/C mouse after pristane induction at month 6. b Incidence of arthritis in Balb/C mice after PBS or pristane injection at month 5 and 6. Mouse numbers per group, 12 in b. Results of b in Fig. S4 were combined data of 2 independent experiments with 6 mice per group. [file 12929_2022_849_MOESM4_ESM.tif]
